# All about portal vein: a pictorial display to anatomy, variants and physiopathology

**DOI:** 10.1186/s13244-019-0716-8

**Published:** 2019-03-21

**Authors:** Carolina Carneiro, Jorge Brito, Carlos Bilreiro, Marta Barros, Carla Bahia, Inês Santiago, Filipe Caseiro-Alves

**Affiliations:** 1 Department of Radiology, Centro Hospitalar Universitário do Algarve EPE, Portimão, Portugal; 2 ABC – Algarve Biomedical Center, Faro, Portugal; 3 Medical Imaging Department, Coimbra Hospital and University Centre, Coimbra, Portugal; 4 Champalimaud Foundation, Lisbon, Portugal; 5 Faculty of Medicine, University of Coimbra and Coimbra Hospital and University Centre, Coimbra, Portugal

**Keywords:** Portal system, Portal vein, Anatomic variation, Venous thrombosis, Portal hypertension

## Abstract

The portal vein (PV) is the main vessel of the portal venous system (PVS), which drains the blood from the gastrointestinal tract, gallbladder, pancreas, and spleen to the liver. There are several variants affecting the PV, and quite a number of congenital and acquired pathologies.

In this pictorial review, we assess the embryological development and normal anatomy of the PVS, displaying selected cases consisting of normal variants, congenital anomalies, and a large and heterogeneous group of acquired conditions that may affect the PV.

## Teaching points

- Portal venous system drains blood from the gastrointestinal tract (apart from the lower section of rectum), spleen, pancreas, and gallbladder to the liver.
- From an embryological point of view, the portal venous system is formed from the 4th to the 12th gestation week, developing from the vitelline venous system in close relation with the umbilical venous system.
- The typical branching pattern of the main portal vein occurs in 65% of individuals in the general population.
- The imaging modality of choice for portal venous system evaluation will depend on the clinical context, patient characteristics, local availability, and expertise.
- Imaging findings of portal vein thrombosis depend on the type of thrombus, degree of thrombosis, extent of collateralization, and age of the thrombus.

## Introduction

Portal venous system (PVS) drains blood from the gastrointestinal tract (apart from the lower section of rectum), spleen, pancreas, and gallbladder to the liver.

The portal vein (PV) is the main vessel of the PVS, resulting from the confluence of the splenic and superior mesenteric veins, and drains directly into the liver, contributing to approximately 75% of its blood flow [1]. Hepatic artery provides the remaining hepatic blood flow. Once in the liver, PV ramifies and reaches the sinusoids, with downstream blood being directed to the central vein at the hepatic lobule level, then to the hepatic veins and inferior vena cava (IVC) to reach the systemic venous system.

**Fig. 1:**
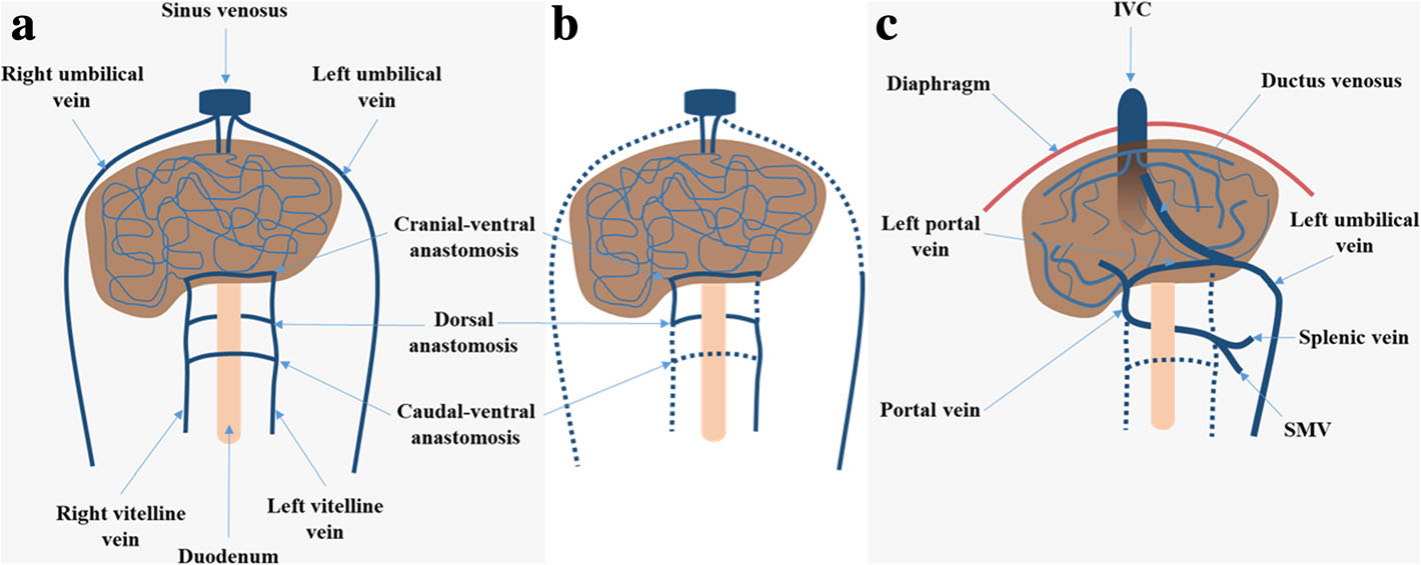
Diagrammatic representation of the embryological development of the PV. **a** The vitelline venous system arrives at the primitive liver as two paired veins (right and left), branches into the hepatic sinusoids, and then coalesce, pierce the *septum tranversum* (primitive diaphragm) and drain into the *sinus venosus* (primitive heart). These two vitelline veins communicate through three pre-hepatic anastomoses around the developing duodenum (cranial-ventral, dorsal, and caudal-ventral). **b** Over time, a selective involution occurs, involving the caudal part of the right vitelline vein, the cranial part of the left vitelline vein, and the caudal-ventral anastomosis. The dorsal and cranial-ventral anastomoses persist and give rise to the main PV and to the left PV, respectively. Initially, the paired umbilical veins lie more lateral than the vitelline ones, and also pierce the *septum tranversum* and drain into the *sinus venosus*. With the development of the liver, the umbilical veins fragment and connect to the hepatic sinusoids. Over time, a selective involution of the right umbilical vein and cranial portion of the left umbilical vein also occurs. **c** The remnant left umbilical vein cranially bifurcates, forming two new communications: one with the IVC through the *ductus venosus*, carrying oxygenated blood from the placenta directly to the fetus; and another with the left PV, supplying directly the liver. After birth, the *ductus venosus* and the left umbilical vein involute and become the *ligamentum venosum* and *ligamentum teres*, respectively

The embryology of the PVS is a complex process that occurs from the 4th to the 12th gestation week, developing from the vitelline venous system in close relation with the umbilical venous system [2] (Fig. 1). Over time, there is a selective involution of the venous network, with the dorsal and cranial-ventral vitelline anastomoses giving rise to the main PV and left PV, respectively [2].

**Fig. 2:**
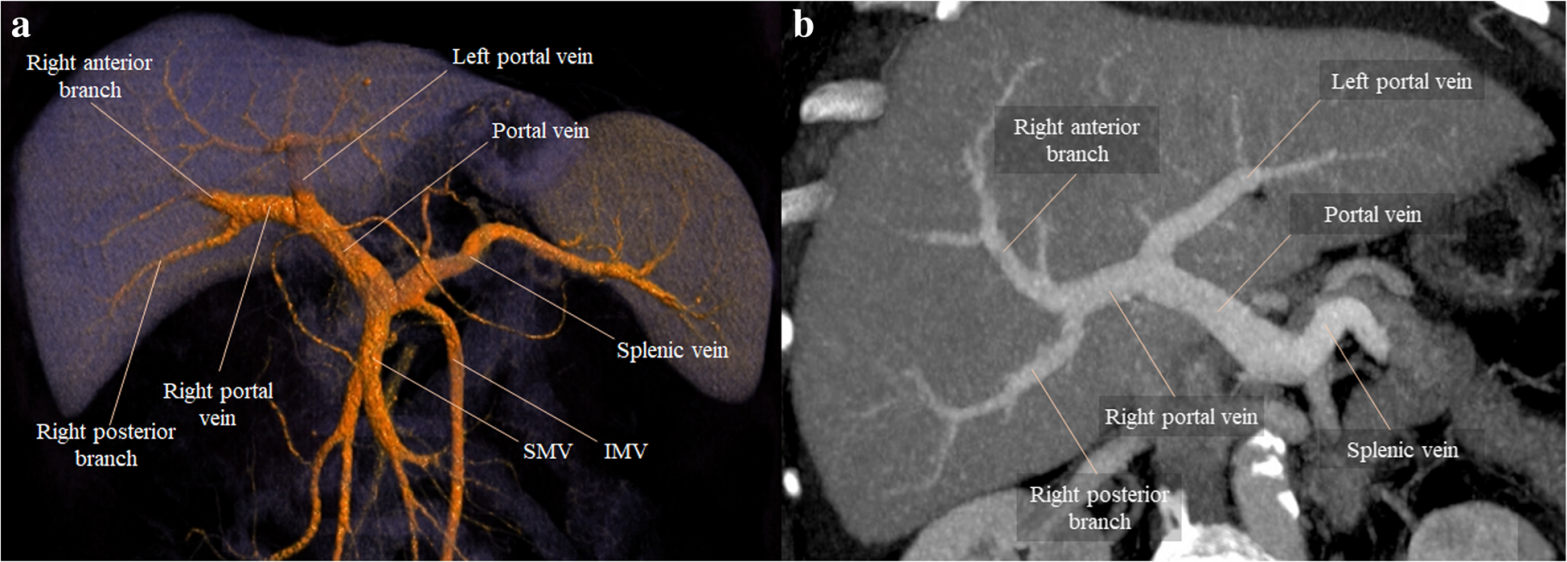
Normal anatomy of the PVS and the typical branching pattern of the main PV. **a** Volume rendering technique (VRT) image from late arterial phase contrast-enhanced CT depicting a global view of the PVS. **b** Maximum intensity projection (MIP) image from portal venous phase contrast-enhanced CT showing the typical branching pattern of the main PV

On normal anatomy, typically, the splenic vein (SV) joins the superior mesenteric vein (SMV) anteriorly to the IVC and posteriorly to the pancreatic neck to form the PV, which ascends within the hepatoduodenal ligament, posteriorly to the hepatic artery and common bile duct, toward the hepatic hilum, where it divides into right and left (Fig. 2a). The left PV is horizontal for a short distance before it turns cranially and branches, supplying Couinaud hepatic segments I, II, III, and IV. The right PV subdivides into anterior and posterior branches; the anterior one supplying segments V and VIII, and the posterior branch supplying segments VI and VII. This typical branching pattern of the main PV occurs in 65% of individuals in the general population [2] (Fig. 2b). Additional tributaries of the PV include the left and right gastric veins, cystic veins, and Sappey veins. The inferior mesenteric vein (IMV) has greater variability, joining the splenic vein (40%), the SMV (40%), or the splenomesenteric confluence (20%) [2].

Every imaging technique has advantages and disadvantages for the noninvasive evaluation of the PVS. The imaging modality of choice will depend on the clinical context, patient characteristics, local availability, and expertise. In most centers, CT is the preferred technique for the evaluation of the PVS, permitting the evaluation of the portal vasculature using high-resolution isotropic acquisition in a short time, and allowing high-quality multiplanar reformations (MPR) and three-dimensional reconstructions [3]. In addition, multiphasic CT allows a comprehensive evaluation of the entire *porta hepatis* with high temporal and spatial resolution. MRI can also be used to evaluate the PVS. The major advantage is the possibility to anatomically evaluate and obtain information about the contents of vascular structures without administering intravenous contrast product and non-using ionizing radiation. However, compared to CT, it is still a more time-consuming, expensive, and less accessible imaging technique, generally with less spatial and temporal resolution necessary to evaluate vascular structures, and also more susceptible to artifacts. Doppler ultrasound is a useful imaging technique in the evaluation of the PVS, is highly available, and the major advantage is allowing a detailed evaluation of the venous flow besides the anatomical information. Sometimes it is conditioned by the biotype and lack of collaboration of the patient, and still have to recur to other techniques if necessary an overall PVS assessment or dynamic contrast information.

Normal PV usually enhances uniformly in the portal venous phase (60–70 s after intravenous contrast administration), measures 11–13 mm in diameter and 7–8 cm in length [3, 4] (Fig. 2b). Since this venous system is valveless, pressure modifications caused by respiration can affect its diameter; therefore, measurements on every imaging technique should be made at deep inspiration, when the caliber is at its greatest [1].

### Anatomic variants and congenital malformations

#### Branching pattern variants

**Fig. 3:**
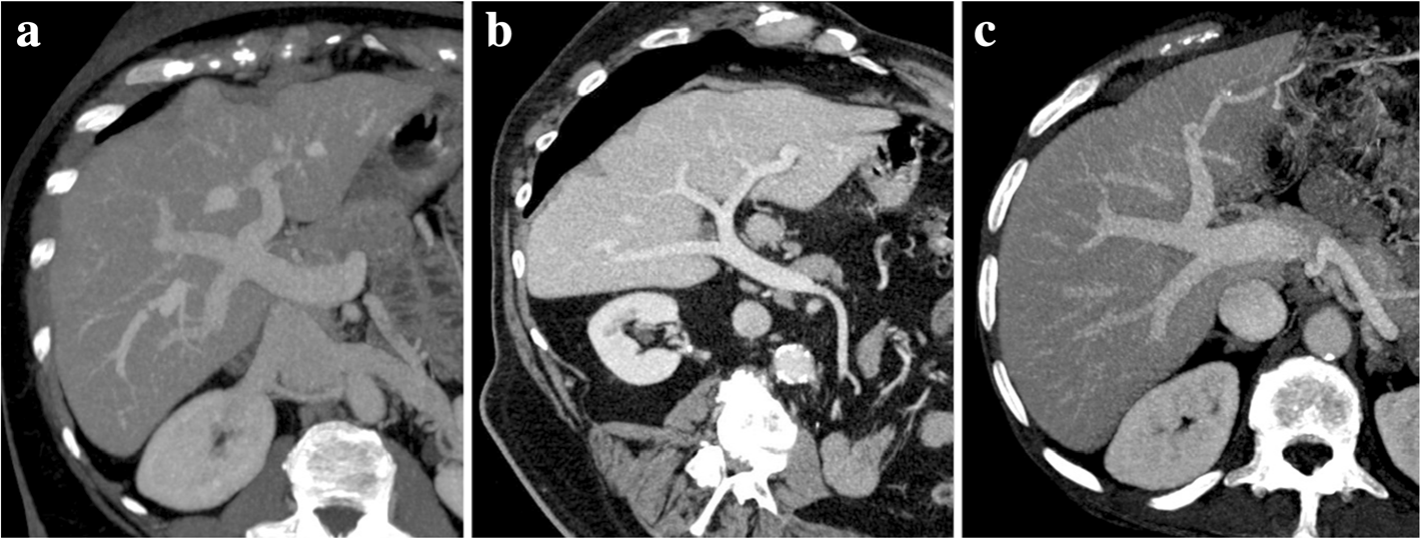
Variants of the PV branching pattern. MIP oblique reconstruction images from portal venous phase CT. **a** PV trifurcation—PV divides into three branches: left PV, right anterior PV, and right posterior PV. **b** Right posterior PV branch arising directly from the main PV. **c** Left PV arising from the right anterior segmental branch

Variants of portal architecture can be found in 20–35% of individuals [1, 5]. Common variant patterns include trifurcation of the main PV (Fig. 3a), right posterior segmental branch arising from the main PV (Fig. 3b), and right anterior segmental branch arising from the left PV [1, 5]. Individual segmental branches arising away from their usual point of origin are also common variants [5]. Other variant branching patterns are less frequently observed, as the left PV arising from the right anterior segmental branch (Fig. 3c), and major anatomic anomalies such as PV duplication and absent branching of the PV are very rare [2].

Awareness of PV branching pattern is important to accurately interpret imaging findings, in planning liver surgery (to ensure that portal perfusion to the future liver remnant is not compromised), liver transplantation (to enable appropriate graft selection so as to avoid complex anastomosis that might compromise the graft or the residual liver in a living donor), and percutaneous interventional procedures.

#### Unusual topography

**Fig. 4:**
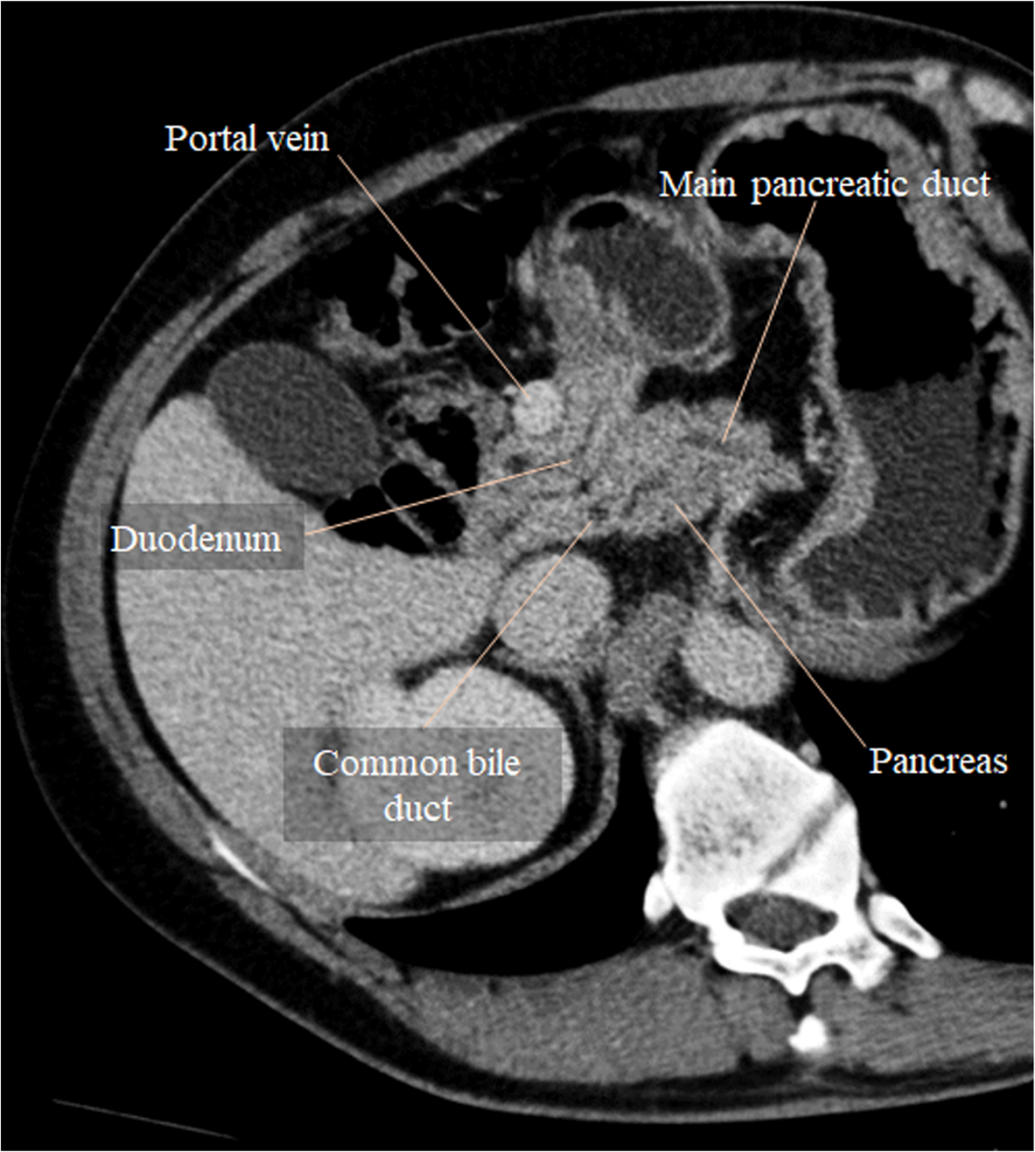
Preduodenal PV. MPR image from portal venous phase CT shows the main PV coursing in front of the duodenum and pancreas, an incidental finding in this case

Normal PV lies posterior to the first part of the duodenum, as it derives embryologically from the dorsal anastomosis of vitelline veins. Preduodenal PV (Fig. 4) is a very rare condition. Its main clinical significance is the frequent association with intestinal obstruction or other congenital malformations, with two-thirds of cases presenting in the first week of life [1, 2].

**Fig. 5:**
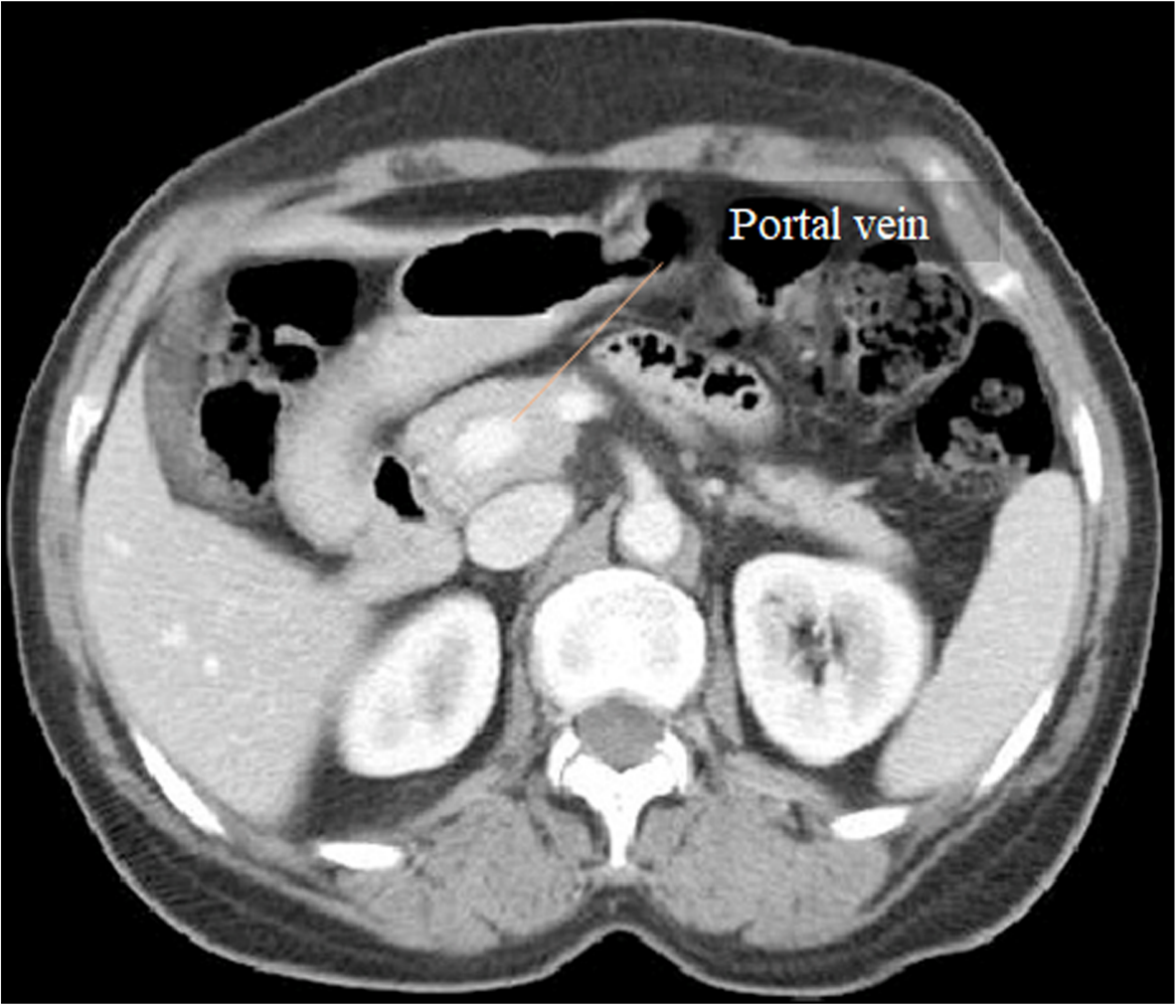
Circumportal pancreas. Portal venous phase CT axial image shows pancreatic parenchyma surrounding PV like an annulus. In this case, it was a suprasplenic type

Circumportal pancreas is a normal anatomic finding in pigs but a relatively uncommon anatomic variant in humans (1–3.4%), usually representing an incidental finding [6]. In circumportal pancreas, the pancreatic parenchyma from the uncinate process is fused with the body of the pancreas, encasing the PV and/or the SMV. It can be classified into three subtypes—suprasplenic, infrasplenic, or mixed, depending upon the level of the pancreatic annulus [6–8]. It can exhibit either an anteportal or an unusual retroportal main pancreatic duct. Combination of an anteportal duct and suprasplenic fusion is the most common subtype [7] (Fig. 5).

#### Congenital portal venous shunts

Portal venous shunts are abnormal communications between portal and systemic venous systems (portosystemic shunts), or between the PVS and the hepatic artery (arterioportal shunts). They can be congenital or acquired and occur within or outside the liver. In this section, we will discuss the congenital ones.

Portosystemic shunts are diversions of portal venous blood into the systemic venous system bypassing the liver. Patients may be asymptomatic, but high-flow shunts are prone to develop hepatic encephalopathy, hepatopulmonary syndrome, and portopulmonary syndrome. Depending on the case, these shunts are managed conservatively, with trans-catheter embolization, or surgery.

**Fig. 6:**
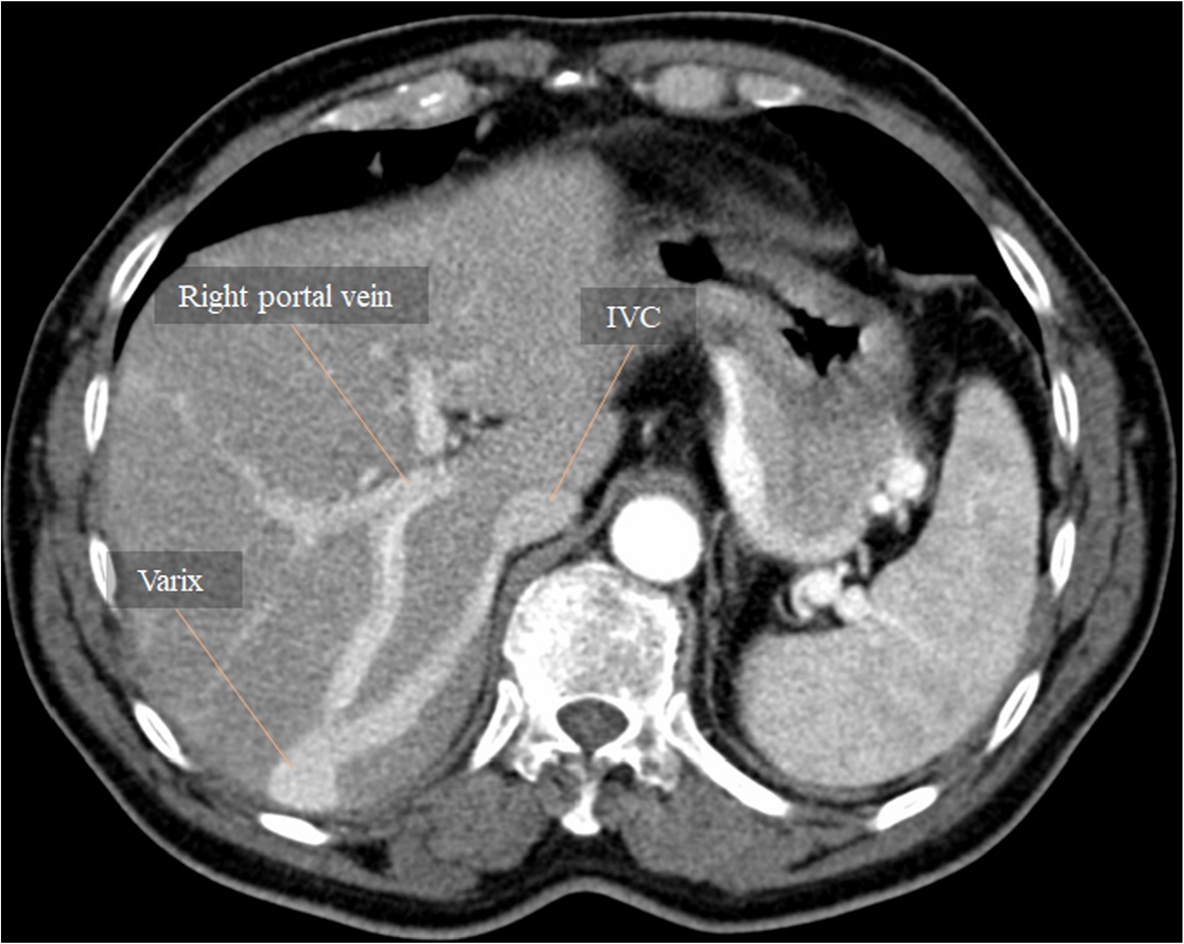
Congenital intrahepatic portosystemic shunt. MPR image from late arterial phase CT displaying the most common type of congenital intrahepatic portosystemic shunt (type 1), with a single vessel connecting the right PV with the IVC. Note the large caliber of both afferent PV branch and efferent hepatic vein, and the presence of a variceal dilatation between both, appearing as a rounded enhancing mass. If considering this focal varix as an aneurysm, we can classify this shunt as a type 3 either

Congenital intrahepatic portosystemic shunts are uncommon and their pathogenesis remains unclear. Interestingly, they are more frequent in the right hepatic lobe [1] (Fig. 6). Four types are classically described, as proposed by Park et al. in 1990 [9] (Table 1). Intrahepatic portosystemic shunts may also be acquired, resulting from trauma (may be iatrogenic) and portal hypertension (PH), as described later.

**Table 1:** Classification of congenital intrahepatic portosystemic shunts based on Park et al. [9]

Type 1	The most common type Single large vessel connects the right PV to the IVC (Fig. 6)
Type 2	Peripheral shunt localized in one hepatic segment, with single or multiple communications between branches of portal and hepatic veins
Type 3	Portal and hepatic veins connected through an aneurysm
Type 4	Multiple communications between peripheral portal and hepatic veins in several segments

**Fig. 7:**
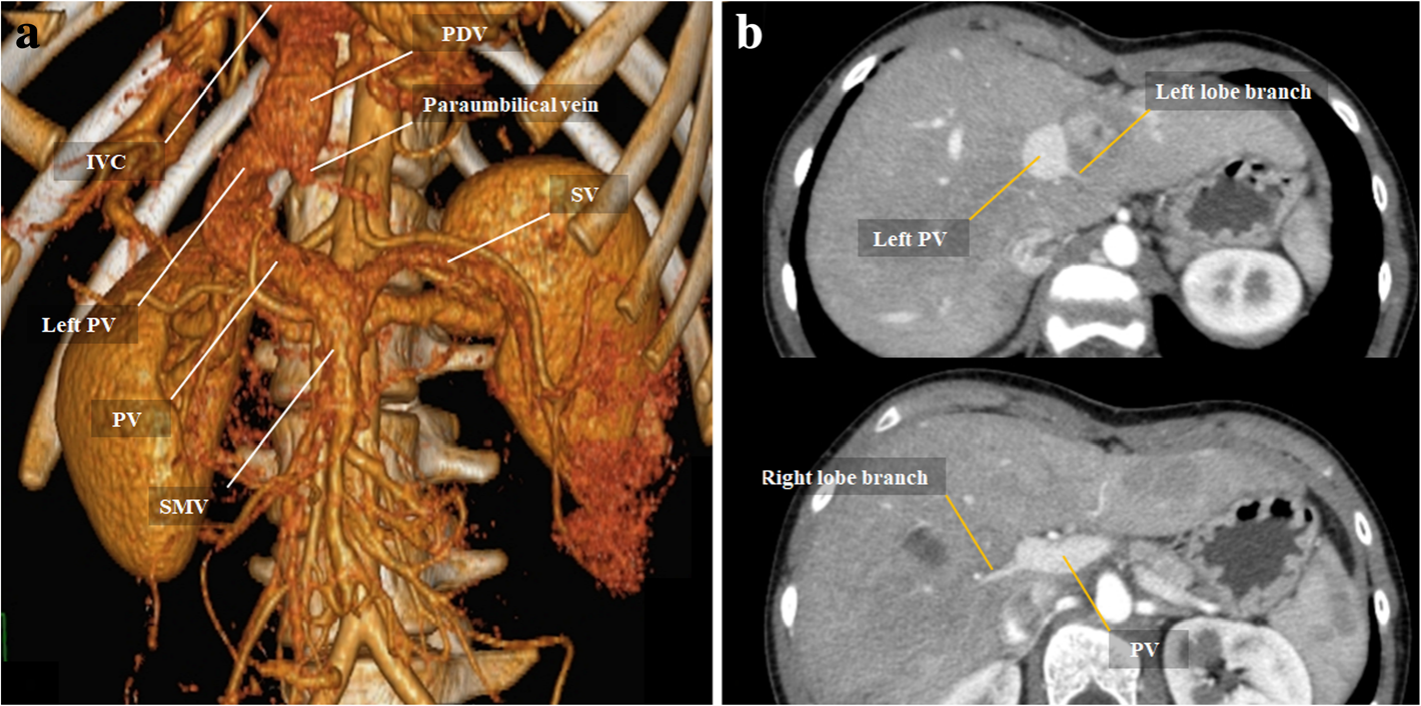
Congenital extrahepatic portosystemic shunt—Abernethy malformation. **a** VRT image from portal venous phase CT presents a type II Abernethy malformation. SV converges with the SMV giving rise to the main PV. After that, the main PV lacks the classic branching pattern into right and left PV. Instead, a large and dominant aberrant left PV reaches the systemic venous system draining into IVC. Taking into account the origin, the route, and the final confluence with IVC, this aberrant left PV-IVC shunt was interpreted as a patent *ductus venosus*. **b** Late arterial phase contrast-enhanced MDCT images demonstrate this was a partial shunting, as we can see small branches arising from the main PV and left PV for both lobes

**Fig. 8:**
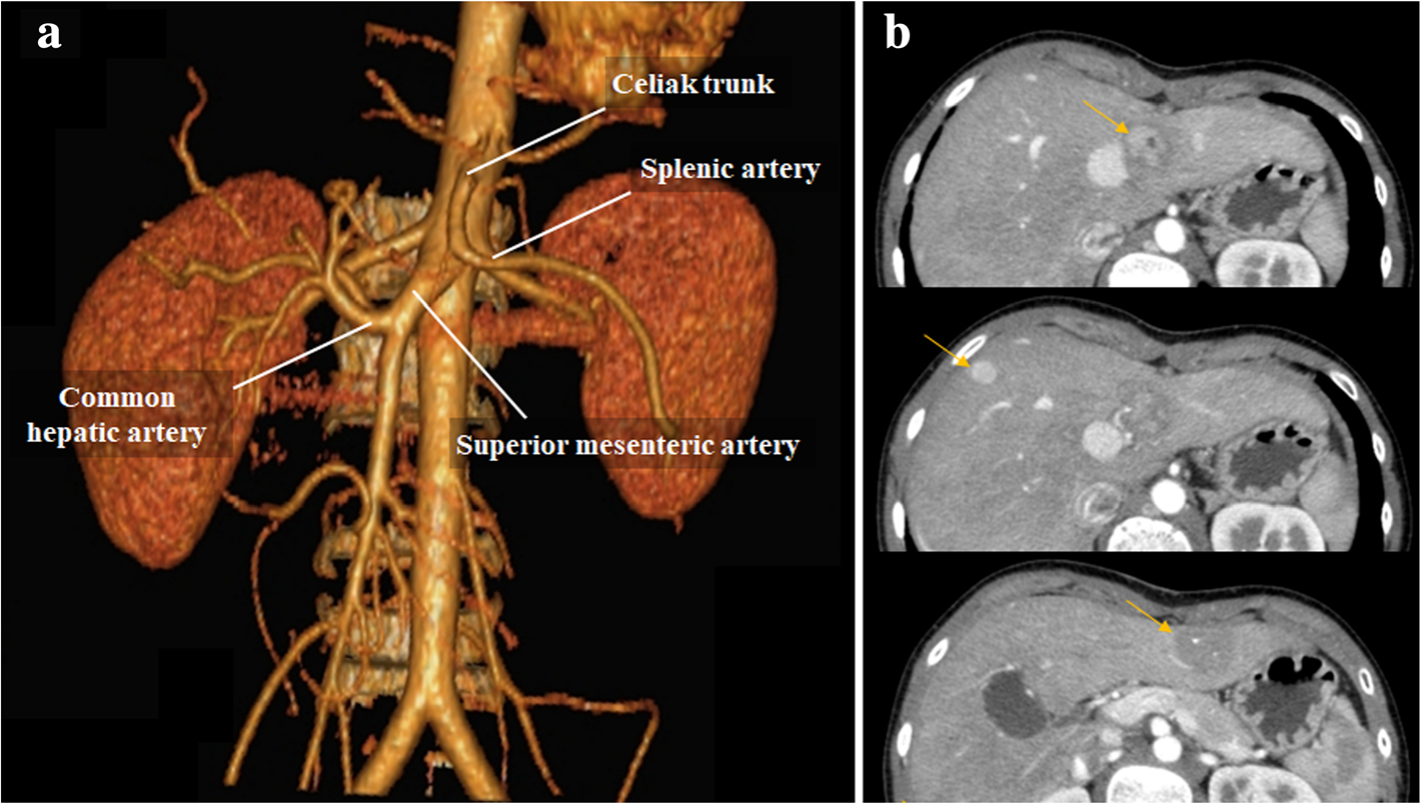
Congenital extrahepatic portosystemic shunt—Abernethy malformation. **a** VRT image from arterial phase CT of the same patient in Fig. 7. Note aberrant origin of the hepatic artery emerging from superior mesenteric artery. There is also increase caliber of the common hepatic artery and its branches, consequence of the augmented arterial compensatory flow. **b** Late arterial phase CT images reveal multiple liver nodules (orange arrows), frequently associated with Abernethy malformation [12]

Congenital extrahepatic portosystemic shunts are rare and represent a group of malformations with high morphological variability, with resultant differences in clinical presentation and optimal operative approach [10]. In 1793, John Abernethy first described a case of a congenital absence of the PV with a portocaval shunt responsible for the portal venous blood diversion [10, 11]. Since 1997, Abernethy malformations became an accepted eponymous to congenital extrahepatic portosystemic shunts, and can be subdivided into two major categories: total shunting with complete absence of intrahepatic portal venous flow—type I; and partial shunting with some preserved hepatic portal venous flow—type II [10, 11]. Nowadays, it is increasingly recognized that there is more a continuum than an absolute distinction between type I and type II. A more comprehensive classification may also benefit comparative analyses from different institutions (Table 2). In both types, most patients suffer premature mortality, associated with the shunting complications and other congenital abnormalities inherent to the syndrome (Figs. 7 and 8) [12].

**Table 2:** Classification of congenital extrahepatic portosystemic shunts—Abernethy malformations [10, 11]

Type	Description
I	Complete absence of intrahepatic portal venous flow
Ia IIb	Congenital absence of the PV with separate drainage of the SMV and splenic vein into systemic veins
	SMV and splenic vein join to form a short extrahepatic PV which drains into a systemic vein
II	Partial shunt with preserved hepatic portal venous flow
IIa IIb IIc	Arising from left or right PV including patent ductus venosus (Fig. 7)
	Arising from main PV
	Arising from the other PV tributaries

Extrahepatic portosystemic shunts as an acquired pathology are the most common portal venous shunts, discussed here later.

Arterioportal shunts may be either congenital (presenting as a fistula or as vascular malformations in hereditary hemorrhagic telangiectasia) or more frequently acquired, as discussed here later.

**Fig. 9:**
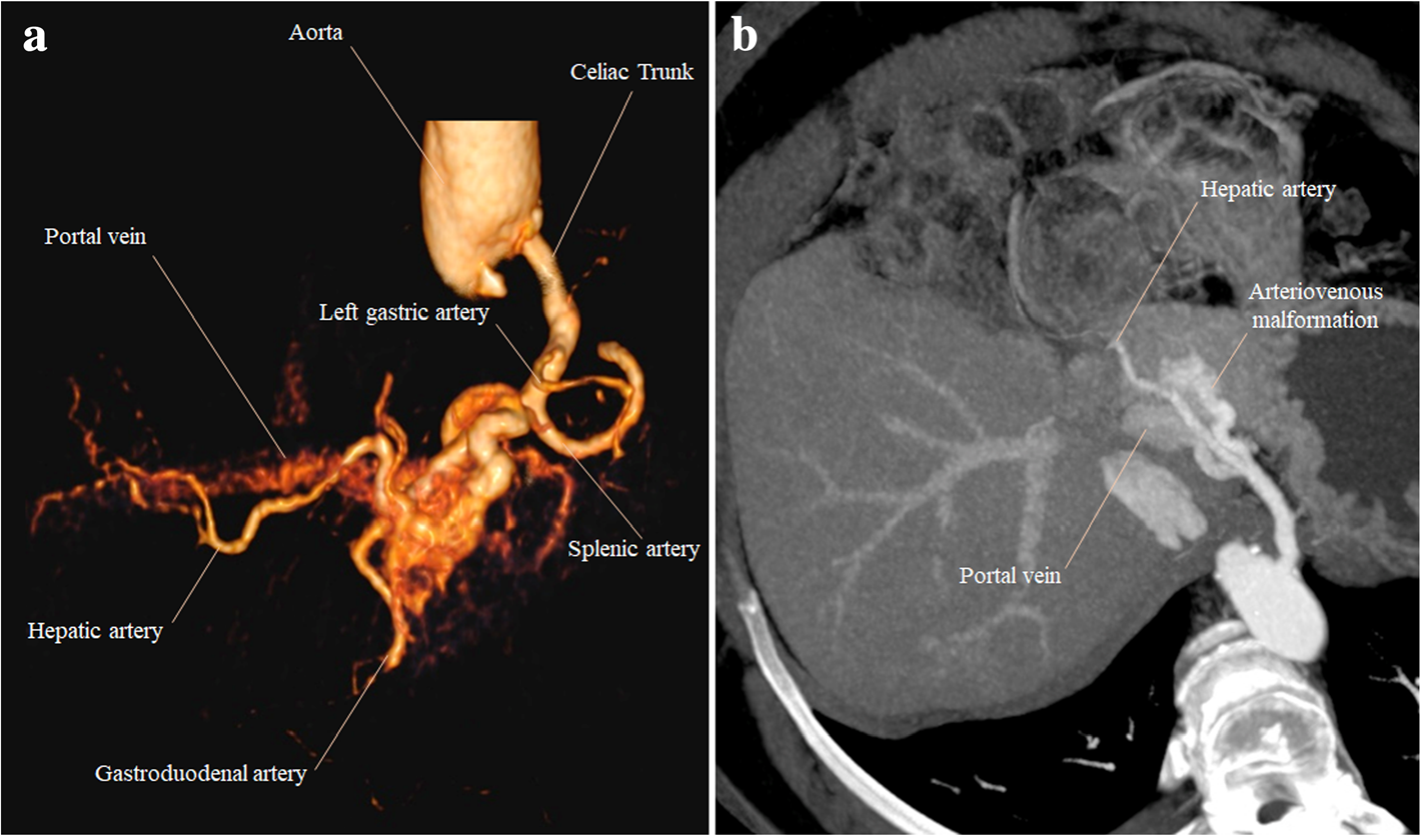
Congenital extrahepatic arterioportal fistula. **a** VRT and **b** MIP images from dominant arterial phase CT. **a** Best depict an early enhancement of the PV in this phase, a sign of arterioportal shunt. **b** Here, we can precisely individualize the arteriovenous malformation, as an aberrant tortuous hepatic artery branch connecting with the left PV, with an attenuation similar to the hepatic artery, filling retrogradely the PV

Congenital arterioportal fistulas are rare and generally solitary connections between hepatic arteries and portal veins (Fig. 9), in contrast to other arteriovenous malformations, which usually have a complex plexiform vascular nidus. As the arterial flow enters the PVS, it follows the lowest gradient pressure, filling retrogradely the PV. High-flow shunts may generate a continuous hepatofugal flow, progressing in time to PH.

**Fig. 10:**
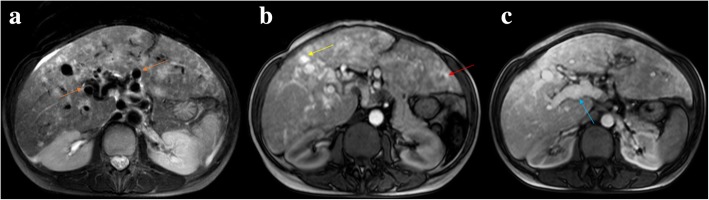
Hereditary hemorrhagic telangiectasia. **a** Axial fat suppressed T2-weighted MR image shows increased caliber of the hepatic artery and its branches (orange arrows). Note also the enlarged liver with heterogeneous texture. **b**, **c** Axial gadolinium-enhanced fat suppressed T1-weighted MR images in the arterial phase show intrahepatic telangiectasias, appearing as rounded small hyperenhancing lesions (**b** yellow arrow). An early contrast filling of portal venous branches is also seen, both peripheral (**b** red arrow) and central branches (**c** blue arrow), in addition to an increased PV caliber

Hereditary hemorrhagic telangiectasia**,** also known as Osler-Weber-Rendu disease, is a rare autosomal dominant trait affecting approximately 1 in 5000 people [13]. It is a multi-organ vascular dysplasia characterized by multiple arteriovenous malformations that lack an intervening capillary network. Liver involvement is characterized by hepatic telangiectasia and usually arteriovenous and less commonly arterioportal, shunts [14]. Arterial phase contrast-enhanced CT typically shows major signs of hepatic arterialization, such as enlarged hepatic arteries, small hyperdense foci representing shunts, and early enhancement of hepatic and/or portal veins (Fig. 10). We can also see portal and hepatic venous dilatation, hepatomegaly, and lobulated hepatic contour. Doppler ultrasound may raise the suspicion of an arterioportal shunt when increased systolic velocity and decreased resistance index of hepatic artery are present, increased velocity of the PV flow, and PV wave inversion demonstrating hepatofugal blood flow.

#### Aneurysms of the portal venous system

**Fig. 11:**
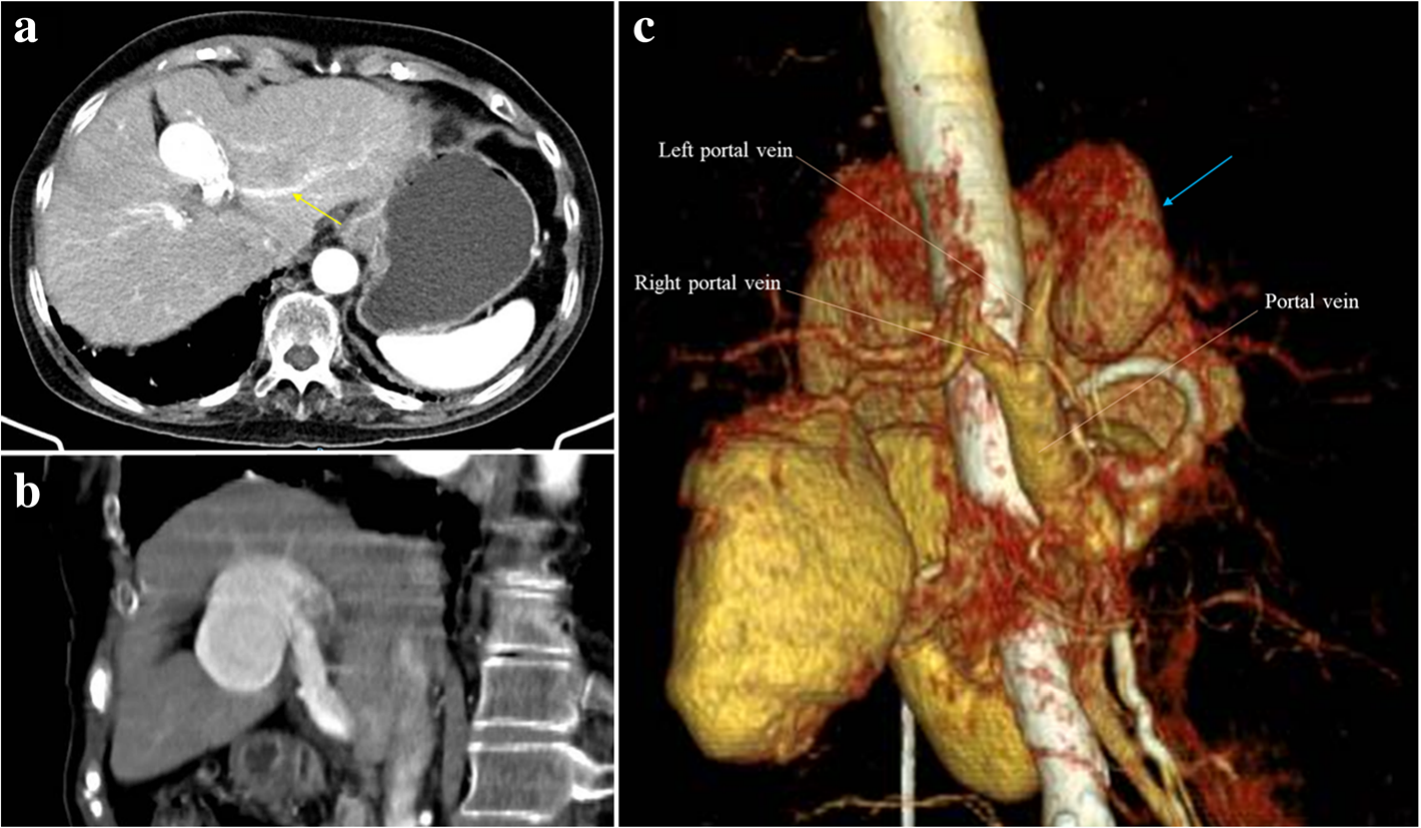
Aneurysm of the PVS. **a**, **b** Axial and oblique MPR images from late arterial phase CT shows an aneurysmal dilatation of left PV with about 3 cm of greater axis. Note the left PV branches arising directly from the aneurysm (yellow arrow). **c** VRT from late arterial phase best depict the origin of the aneurysm in the PVS

Aneurysms of the PV are rare and represent only 3% of all aneurysms of the venous system [15]. Most patients are asymptomatic and both congenital and acquired causes are proposed. Most common locations are the splenomesenteric venous confluence, main PV, and intrahepatic branches at bifurcation sites (Fig. 11) [15].

### Acquired portal vein pathologies

#### Portal vein thrombosis

Portal vein thrombosis, also known as pylethrombosis, is uncommon, although it can be associated with a number of common clinical conditions including cirrhosis, intraabdominal inflammation, hypercoagulability states, abdominal trauma, and iatrogenic complications [2, 3]. In PV thrombosis, imaging appearance depends on the degree and chronicity of the thrombosis, as well as the extent of collateralization, but typically imaging findings show a complete or partial filling defect within the portal venous lumen on contrast-enhanced imaging.

**Fig. 12:**
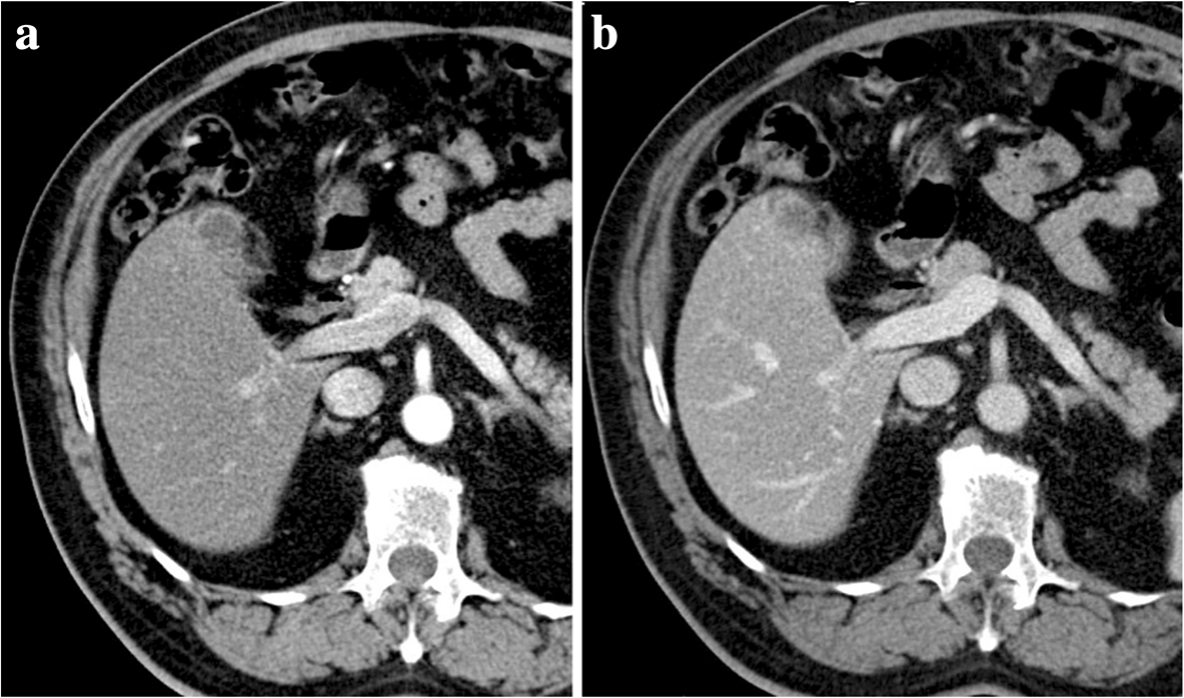
Pseudothrombosis phenomena. **a** Late arterial phase CT axial image shows a heterogeneous low-attenuation PV content, due to pseudothrombosis phenomena. **b** Accordingly, on portal venous phase, the PV shows homogeneous fulfilling, discarding a hypothesis of a true thrombosis

The reader must be aware that a PV pseudothrombosis may be occasionally depicted, consisting in a low-attenuation filling defect-like appearance in the main PV lumen resulting from the mixing of the enhanced splenic vein flow with the non-enhanced SMV flow during the late arterial phase or early portal venous phase. However, contrarily to a real thrombus, this pseudo-filling defect disappears on portal venous phase (Fig. 12).

**Fig. 13:**
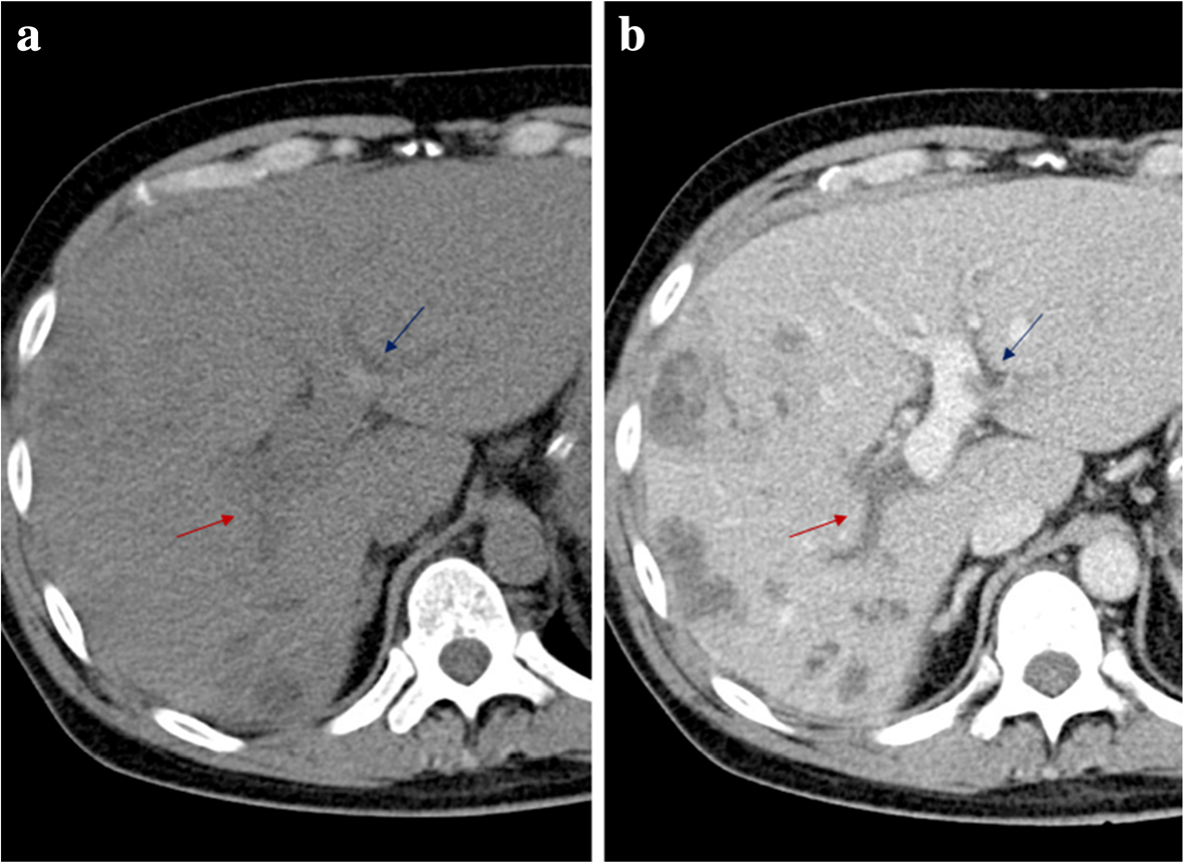
Acute vs. subacute/chronic PV thrombosis. **a** Plain CT axial image shows high-attenuation content within a left PV branch (blue arrow)—acute thrombus. **b** Portal venous phase CT axial image clearly reveals an additional low-attenuation filling defect in the right PV, extending into its right posterior branch (red arrow)—subacute/chronic thrombus

**Fig. 14:**
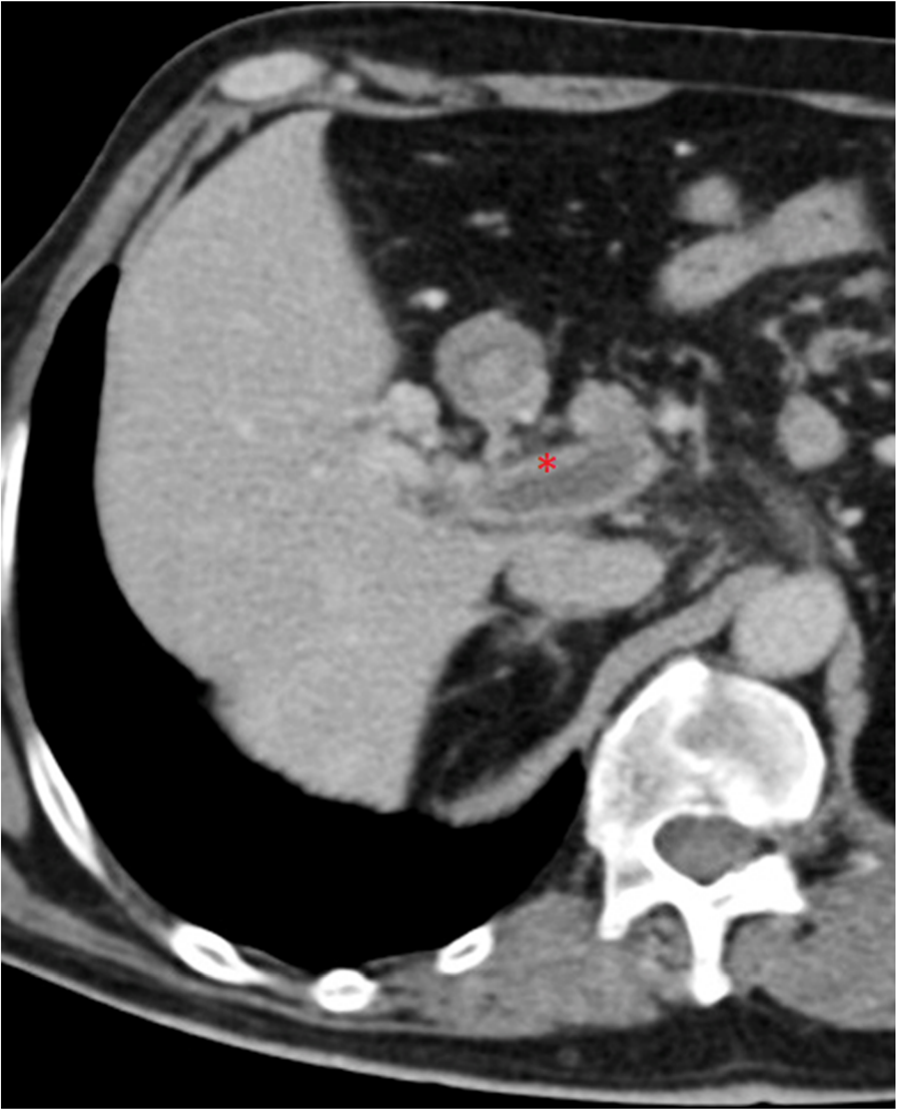
Acute portal vein thrombosis. MPR image from portal venous phase CT shows a luminal thrombus within the main PV, and enhancement of the vessel wall (red asterisk), presumably due to still normal flow through its *vasa vasorum*

Acute PV thrombosis may have different clinical presentations, ranging from the asymptomatic patient, nonspecific abdominal pain, to deteriorating PH with increased risk of variceal bleeding and shock [2, 3]. Acute thrombus usually presents as a high-attenuating luminal defect on plain CT (Fig. 13a), in contrast to the low-attenuating defect usually observed on subacute/chronic stages. Despite the thrombus age, in portal venous phase, it always appears as a luminal filling defect (Fig. 13b). Sometimes, at its early stages, despite the lack of enhancement of the PV lumen, enhancement of the vein wall may occur, thought to represent either dilated *vasa vasorum* or a patent thin peripheral lumen [15] (Fig. 14).

**Fig. 15:**
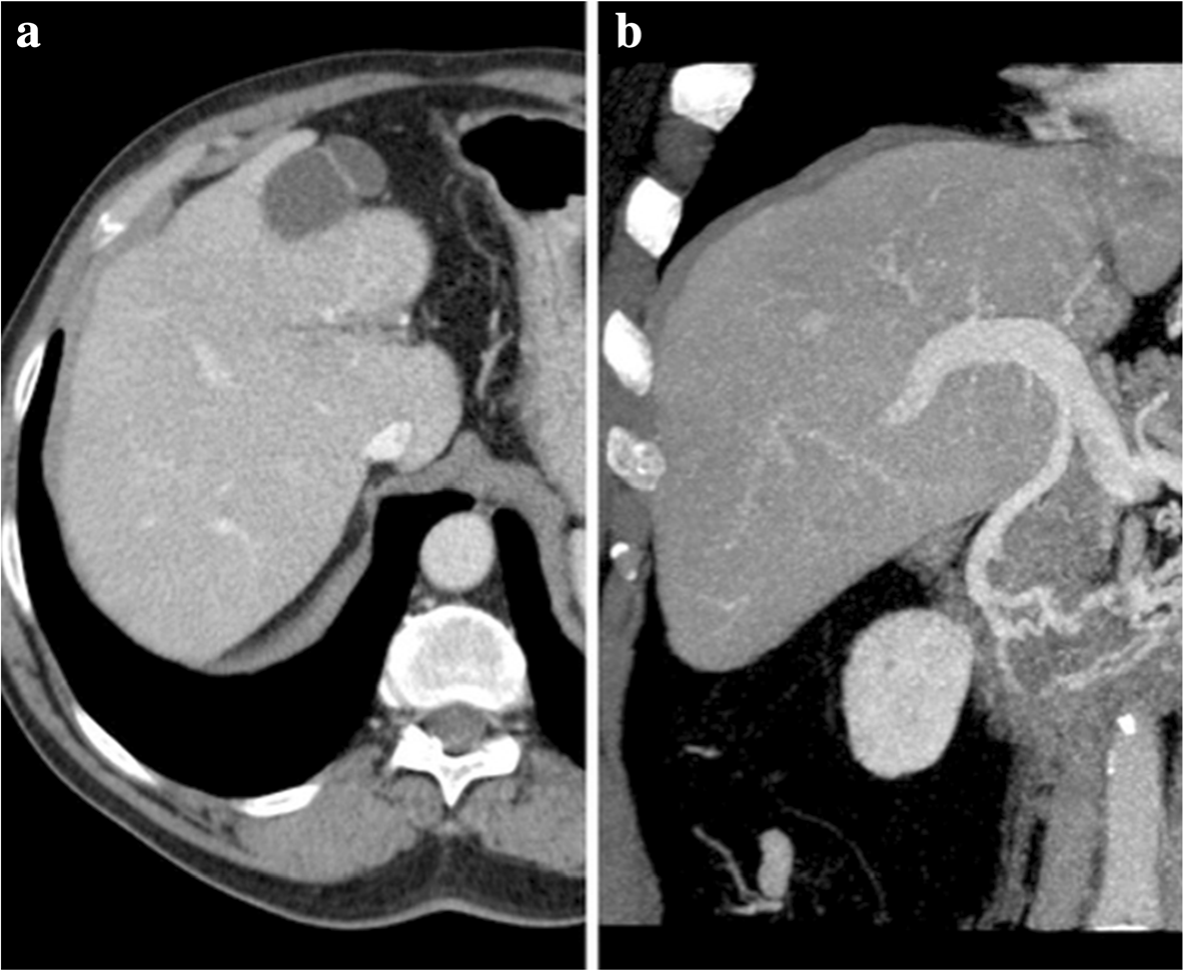
Portal vein thrombosis, chronic evolution. **a** MPR and **b** MIP images from portal venous phase CT show the evolution of a previous left PV complete thrombosis. With time, the unsupplied hepatic segments totally disappeared with atrophic involution of the left lobe

Additional CT imaging findings of subacute and chronic PV thrombosis include transient hepatic enhancement differences (THED), cavernomatous transformation of the PV, and total disappearance of the thrombosed segment with involution of the reliant structures (Fig. 15).

**Fig. 16:**
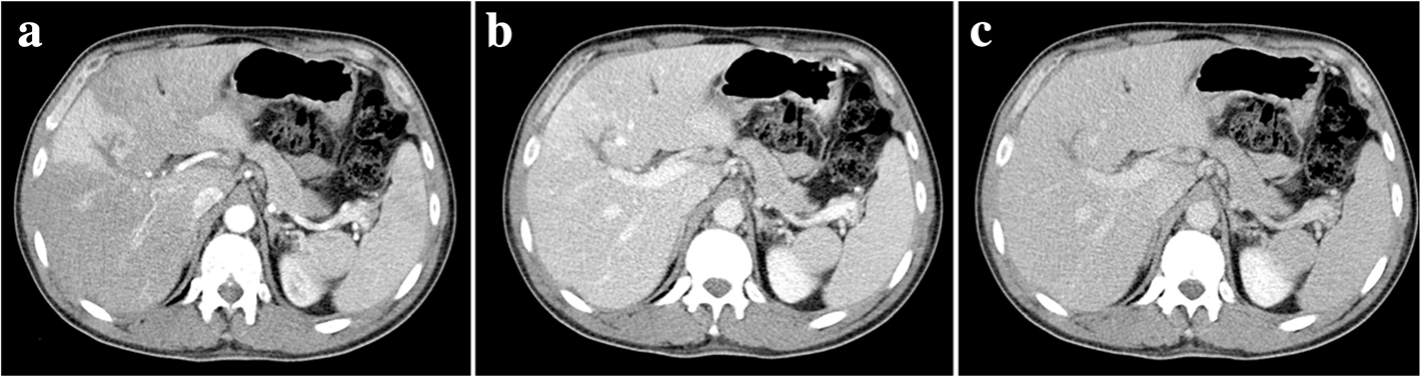
Portal vein thrombosis with THED. **a** Arterial phase CT axial image shows a triangular-shaped area of arterial enhancement, corresponding to the parenchyma formerly supplied by an occluded right anterior PV branch. At portal venous (**b**) and late phases (**c**) the hepatic parenchyma homogenizes, and there is persistent absence of venous enhancement due to bland thrombus

THED is the end result of the decreased portal venous flow to a liver segment or region and subsequent buffer response of the arterial counterpart via the opening of the physiological distal arterioportal shunts. Thus, the arterial flow takes the role and is responsible for keeping the blood supply to that segment/region, in contrast to the remaining liver, that continues to receive the vast majority of its blood supply from PVS. The net difference results in arterial hyper-enhancement of the involved segment/region, in contrast to the low-attenuating remaining parenchyma. The perfusion abnormality is no longer recognized in the portal venous and/or equilibrium phases of the dynamic study (Fig. 16).

**Fig. 17:**
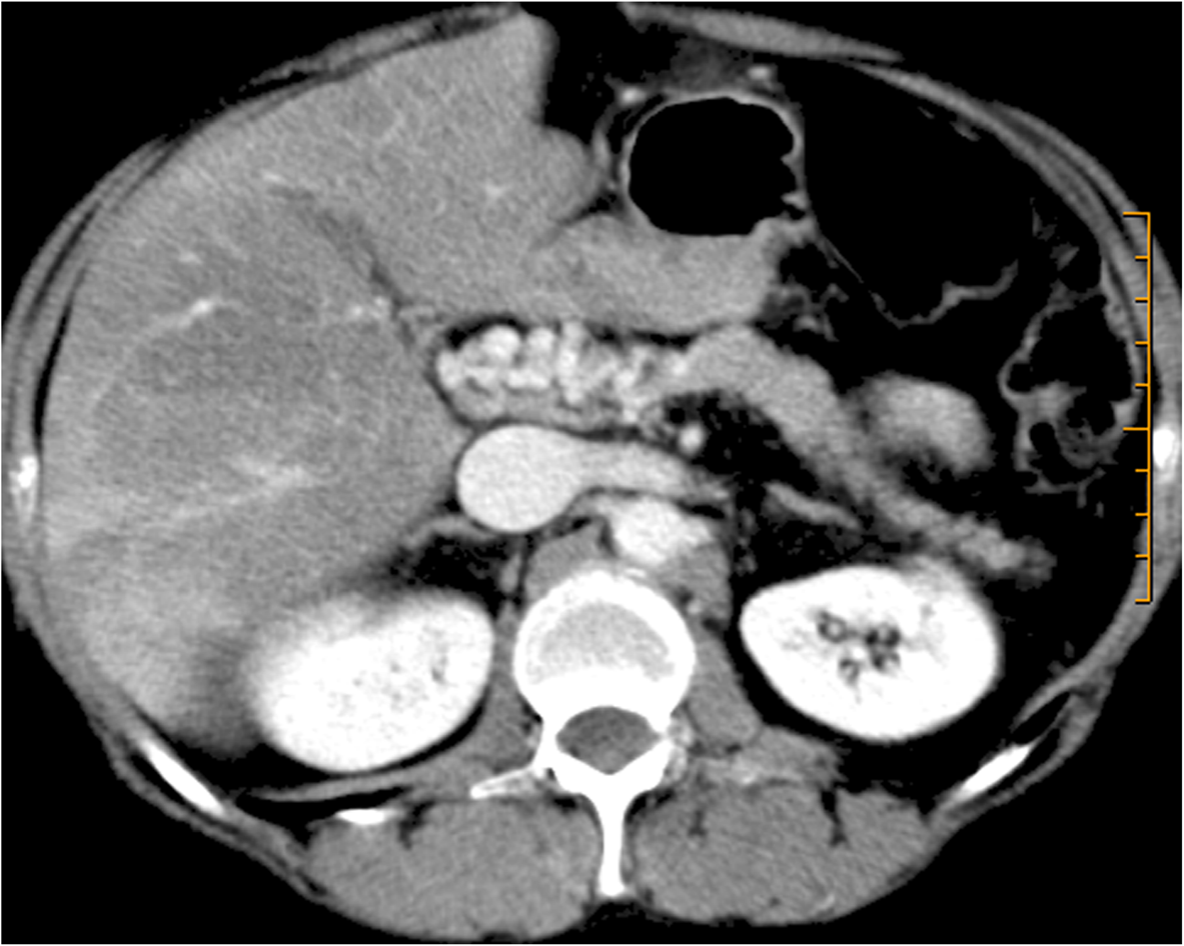
Cavernomatous transformation of PV. Late arterial phase CT shows absence of a well-defined PV and multiple enhancing veins instead, representing portoportal collateral vessels—portal cavernoma. Note the THED associated with cavernoma

**Fig. 18:**
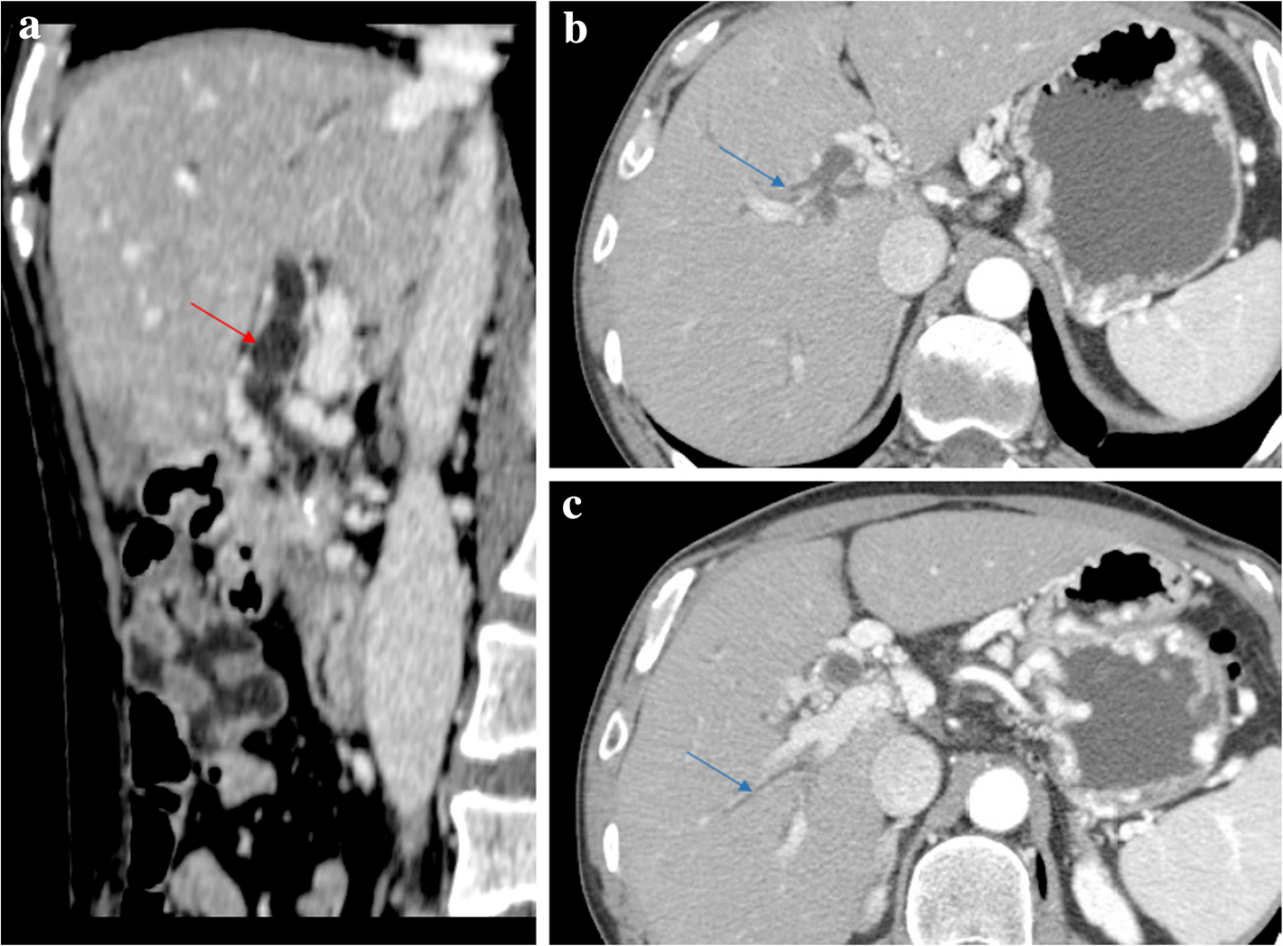
Portal biliopathy. Sagittal portal venous phase CT images shows tortuous common bile duct (red arrow) encircled by portal cavernoma, leading to dilation of peripheral bile ducts (blue arrows). Note also gastric varices due to PH

Cavernomatous transformation of PV consists on the development of multiple venous channels within and around a previously stenotic or occluded PV, acting as portoportal collateral vessels. Dilated cystic veins, epicholedocal plexus (of Saint) and paracholedocal plexus (of Petren), dilated gastric venous branches (left and right gastric veins), and the stenotic/occluded main PV itself may also form the portal cavernoma. This transformation can occur as soon as 6–20 days after a thrombotic event, even if partial recanalization of the thrombus develops [15]. The veins are usually insufficient to maintain the normal portal pressure and thus other signs of PH eventually develop. THED are frequently observed, because the central regions of the liver are better supplied by the cavernomatous PV than the peripheral ones. The portal venous flow decrease at the periphery of the liver causes again an arterial compensatory buffer response and inhomogeneous, peripheral, patchy areas of transient high attenuation are recognized in the arterial phase (Fig. 17). Sometimes, cavernomatous transformation of PV can encircle the common bile duct at porta hepatis, causing biliary obstruction with subsequent dilatation of the biliary tree—an entity described as portal biliopathy (Fig. 18).

**Fig. 19:**
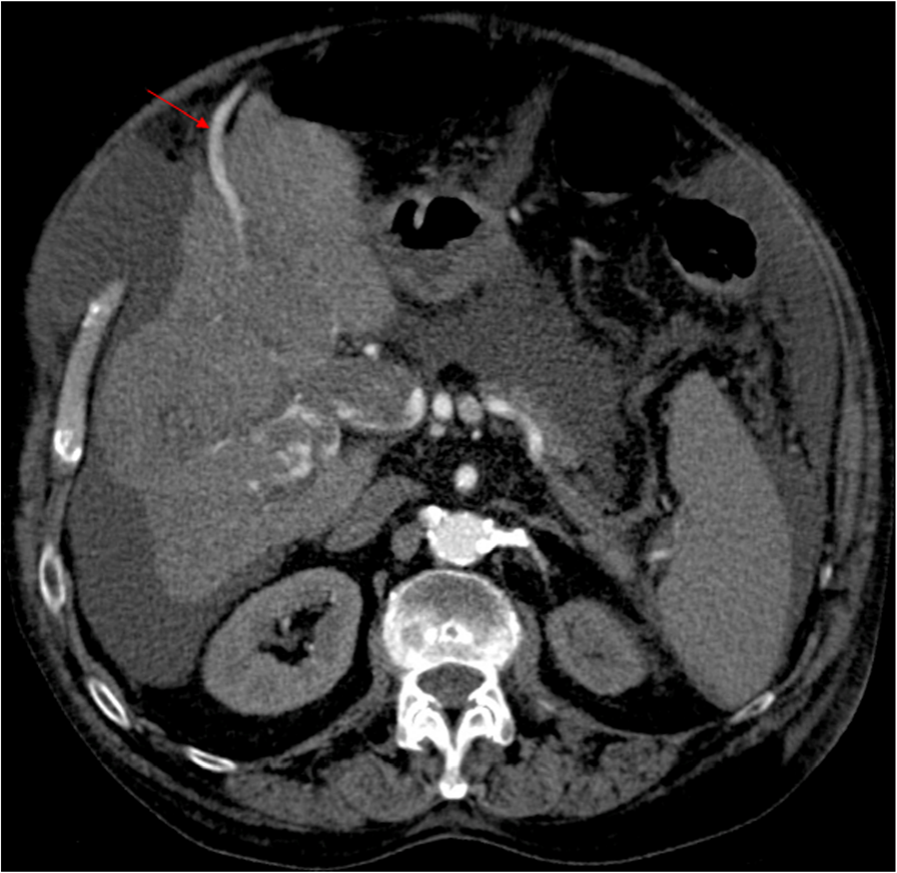
Tumoral PV thrombosis. MPR image from early arterial phase CT shows heterogeneous enhancing PV thrombus, despite absent enhancement of both splenic and SMV (not shown). Note also the presence of cirrhosis (favoring hepatocarcinoma as the cause of the thrombus), ascites, and repermeabilization of the paraumbilical vein (red arrow), signs of PH. The early enhancement of the recanalized paraumbilical vein also suggests the presence of malignant arterioportal fistula

Sometimes, it is difficult to differentiate a bland PV thrombus from a malignant thrombus, and this differentiation is critical since malignant thrombi are associated with a poorer prognosis, and have huge impact on patient management. The most useful imaging finding to distinguish between malignant from bland thrombi relies on the demonstration of heterogeneous contrast enhancement of the tumoral thrombus, especially seen at the arterial phase. Additional findings are contiguity of the thrombus with a tumor (most frequently a hepatocellular carcinoma) and vessel dilatation due to the tumoral casts growing inside. Malignant portal venous thrombus may also present as an arterioportal fistula, as arterial flow enters the PVS following its low gradient pressure, retrogradelly filling the PV or collateral vessels (Fig. 19).

#### Portal venous gas

**Fig. 20:**
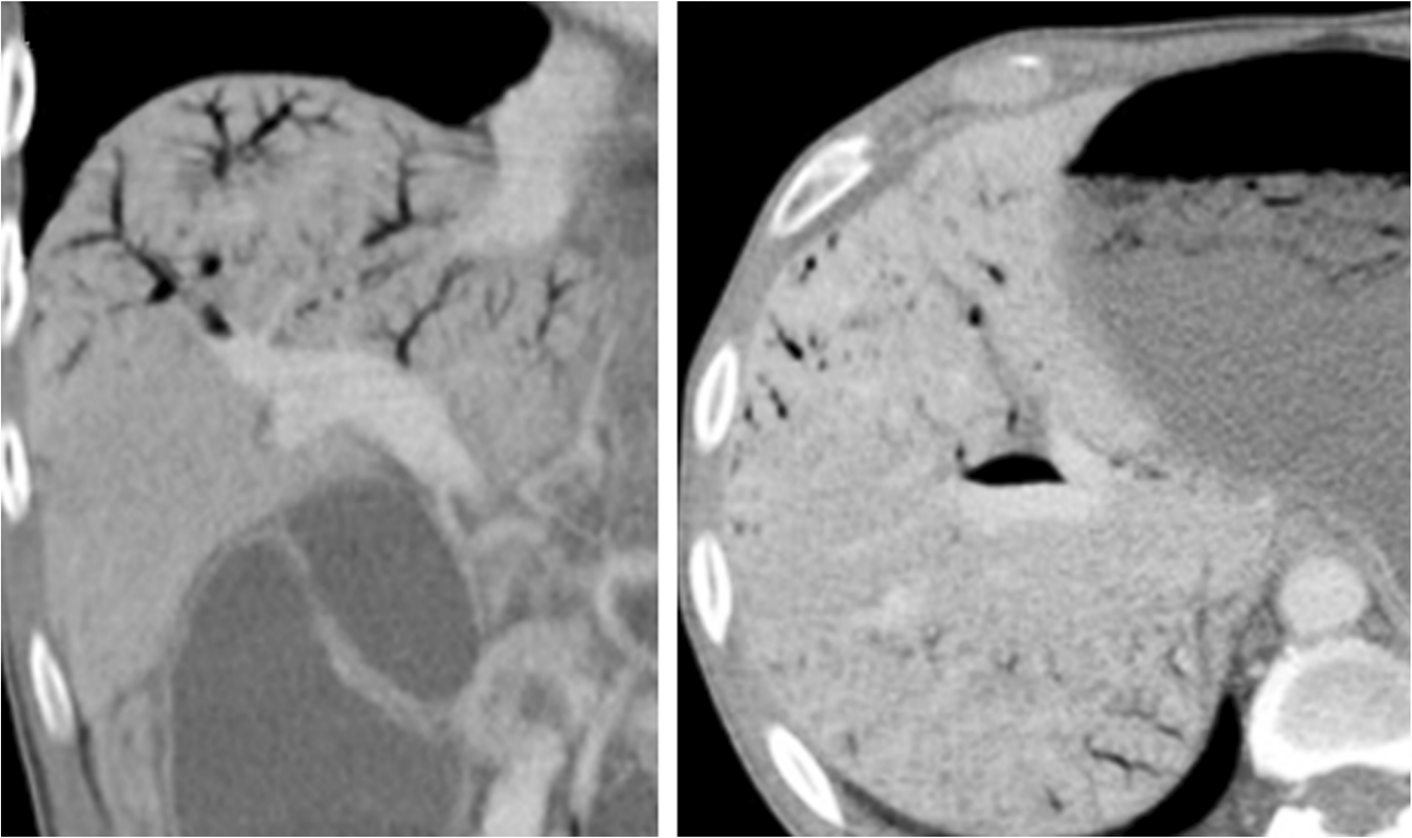
Portal venous gas. MPR images from portal venous phase CT shows air branching pattern fulfilling some segments of peripheral right PV branches

Portal venous gas (aeroportia) was traditionally considered a life-threatening sign thought to be a finding almost exclusive of advanced mesenteric ischemia. Nowadays, other recognized causes of portal venous gas include inflammatory bowel disease, diverticulitis, bowel distention, intra-abdominal sepsis, trauma, iatrogenic, and idiopathic causes. Imaging findings on CT relates to a linear, branching pattern, or ovoid areas with gas attenuation, within the main PV or its branches (Fig. 20). As aeroportia needs to be distinguished from pneumobilia (gas in bile ducts), it has to be taken into account the pattern of gas distribution: peripheral in portal venous gas, even reaching the sub-capsular areas, and central in pneumobilia, following the direction of the portal venous flow and biliary drainage, respectively.

#### Portal hypertension

Clinically significant PH is defined as a portal venous pressure above 10 mmHg, and it is associated with an increased risk of developing varices and variceal bleeding [4, 14, 16].

**Fig. 21:**
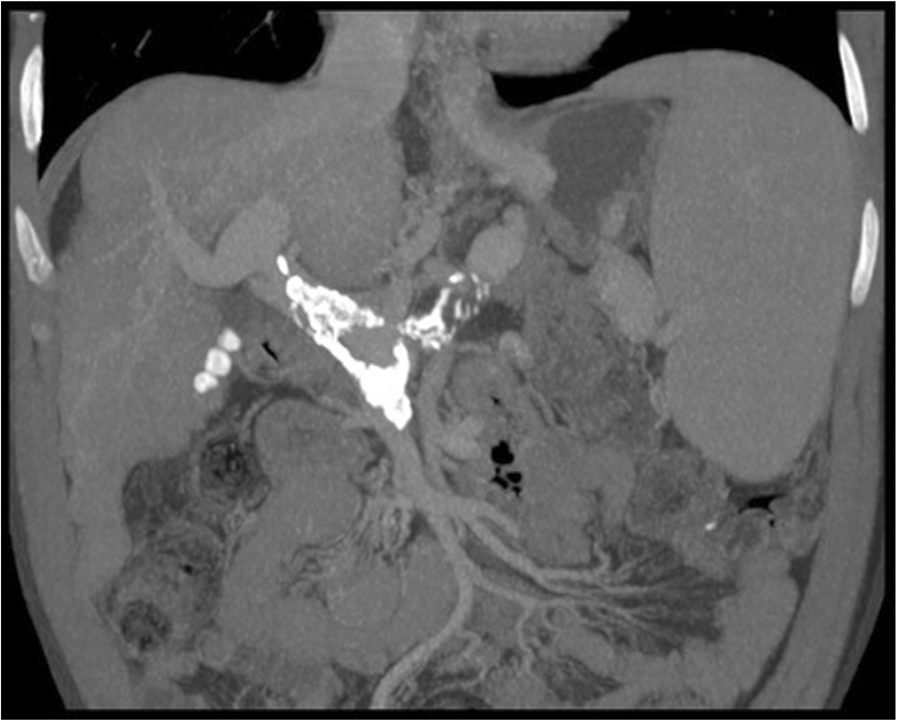
Signs of PH. MIP image showing extensive calcification of PV and its tributaries, in a patient with a long-standing PH. Note the presence of splenomegaly, also a sign of PH, and the incidental finding of gallbladder stones

**Fig. 22:**
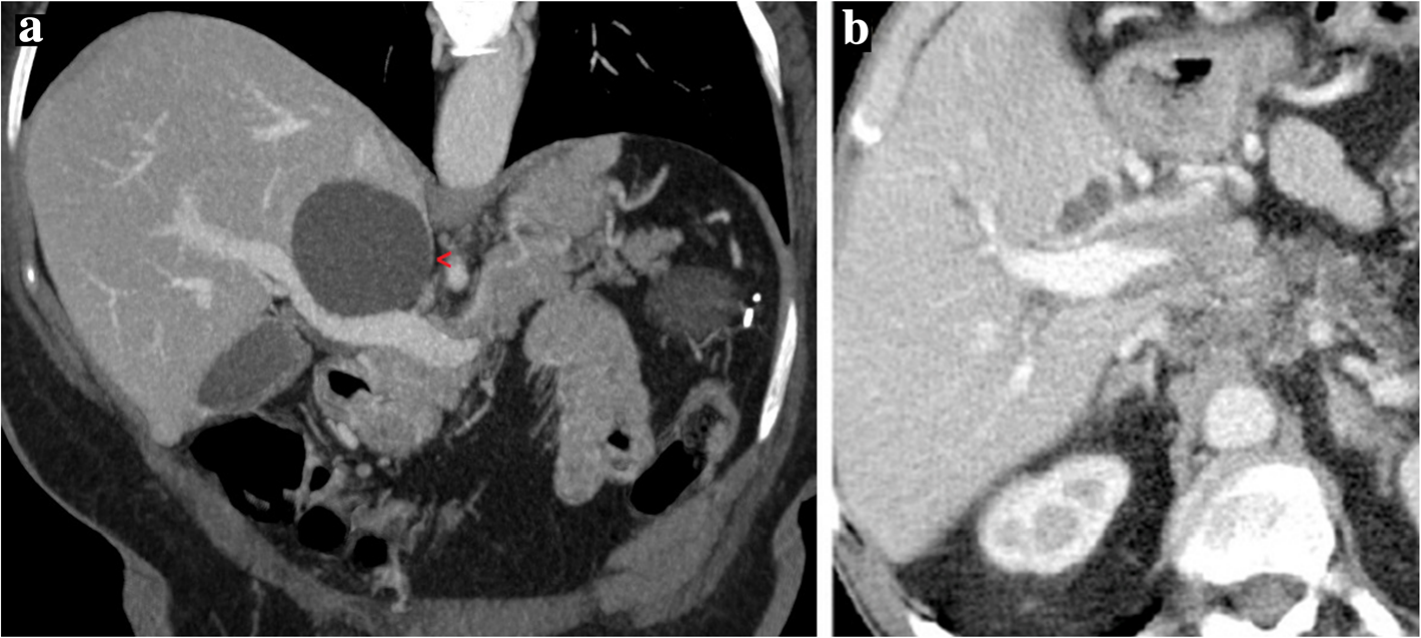
Pre-sinusoidal causes of PH. **a** MIP image from portal venous phase CT shows extrinsic compression of PV by a large hepatic cyst (red arrow head), a benign cause of pre-sinusoidal PH. **b** MPR image from portal venous phase CT show PV invasion by a pancreatic adenocarcinoma, a malignant cause of pre-sinusoidal PH

When pressure increases, PVS rearranges, and one of the first signs is the increase of veins caliber: PV (> 13 mm), SMV, and splenic vein (> 10 mm) [14]. One should note that no significant venous dilatation might be present in PH in case of compensatory portosystemic collateral pathways. In the initial stages of PH increased diameter and tortuosity of hepatic artery and its branches may also be seen, as a result of hepatic venous inflow disturbance. Along with these findings, splenomegaly (Fig. 21) is frequently present, considered when splenic bipolar diameter is increased (> 130 mm in males and > 120 mm in females) [8]. In more advanced cases, ascites may occur as a result of decompensated PH. PVS calcification has a strong association with long standing PH, regardless of the underlying etiology (Fig. 21). Causes of PH can be easily identified, and categorized according to its relation to the hepatic sinusoids: pre-sinusoidal [e.g., PV thrombosis, extrinsic compression of PV (Fig. 22a), tumor invasion (Fig. 22b)]; sinusoidal (e.g., cirrhosis, sinusoidal obstruction syndrome—previously known as hepatic veno-oclusive disease); and post-sinusoidal (e.g., Budd-Chiari syndrome).

Obliterative portal venopathy (OPV), also known as idiopathic PH and hepatoportal sclerosis, is a major cause of non-cirrhotic PH [17]. Its etiology and pathogenesis remain unclear, but primary lesions remain in portal tracts, with varying degrees of thrombosis, fibrosis, and sclerosis [17, 18]. Typically, the hepatic synthetic function is preserved, and the laboratory evaluation reveals only mild nonspecific hematological abnormalities, mostly related to hypersplenism. Another clue to the diagnosis is its common association with a range of diseases, the most common being prothrombotic diseases, immune-mediated disorders, and human immunodeficiency virus infection. OPV diagnosis remains a challenge and patients are often misdiagnosed as cryptogenic cirrhosis. At present, the diagnosis of OPV is based on pathologic findings, excluding cirrhosis and other causes of PH. This differentiation is crucial because patients with OPV usually have preserved liver function, and patient management differs from other causes of PH.

**Fig. 23:**
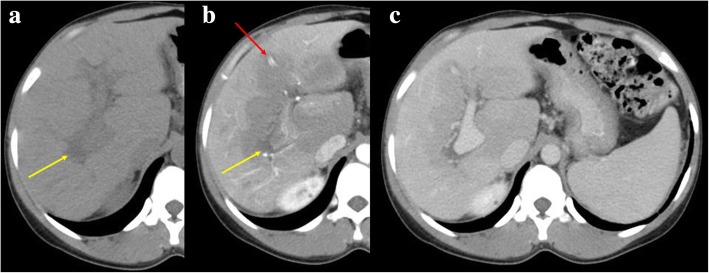
Obliterative portal venopathy. 32-year-old female, with HIV infection and slightly increased bilirubin levels, who complained of increased abdominal volume. Axial (**a**) plain, (**b**) arterial, and (**c**) portal venous phase contrast-enhanced CT images show some morphologic liver changes, with hypertrophy of the caudate lobe, but regular contours and no atrophy of segment IV. Right PV is hypoattenuating before and after contrast material administration (yellow arrows), a feature suggesting non-acute thrombus. Hepatic parenchyma displays heterogeneous peripheral enhancement in arterial phase (**b**) due to compensatory increased arterial flow. This THED fades in portal venous phase (**c**). Some PH stigmata are present, as such splenomegaly, permeable umbilical vein (red arrow), and other varices. Liver biopsy was performed and OPV diagnosis pathologically confirmed

OPV imaging findings comprise the similar signs described for other general causes of PH but the most useful features are the disparity in caliber from the central PV branches (increased) and its first and second order segmental branches (clearly reduced or even not detectable) (Fig. 23). Nodular regenerative hyperplasia (focal nodular hiperplasia-like nodules) and perfusion disorders are also more frequent in cases of OPV than in cirrhosis. Considering the differential diagnosis, it should also be noted that the combination of hypertrophy of the caudate lobe, atrophy of segment IV, and a nodular liver surface are signs more often associated whit true cirrhosis but not with OPV.

Despite the PH etiology, acquired extrahepatic portosystemic shunts may develop over time in an attempt to reduce the hepatofugal venous flow (discussed below).

#### Acquired portosystemic shunts

Acquired extrahepatic portosystemic shunts are the most common shunts among PVS. A large group of pathologies can trigger its development, but the most common cause is PH [2, 15]. Other causes include splenic, splenomesenteric, and SMV stenosis or obstruction. Hepatopetal venous flow is rerouted away from the liver (hepatofugal) through collateral pathways to reach the low-pressure systemic vessels. More than 20 pathways have been described, the most common being gastroesophageal, para­esophageal, paraumbilical, splenorenal (mostly left-sided), and inferior mesenteric venous collateral vessels, in order of decreasing frequency [2, 15]. The imaging findings are the presence of dilated tortuous veins in relation with the PVS and systemic venous system. Can be found on ultrasound, on CT are best depict at portal venous phase as tubular enhancing structures and on MRI as flow voids.

Coronary collateral veins at the lesser omentum are the most frequently depicted varices [5] (Fig. 18c). Their presence has a high sensitivity rate to PH diagnosis. Gastric varices are commonly located in the posterosuperior aspect of the gastric fundus (Fig. 18c), and drain into the esophageal or paraesophageal veins, or occasionally into the left renal vein.

**Fig. 24:**
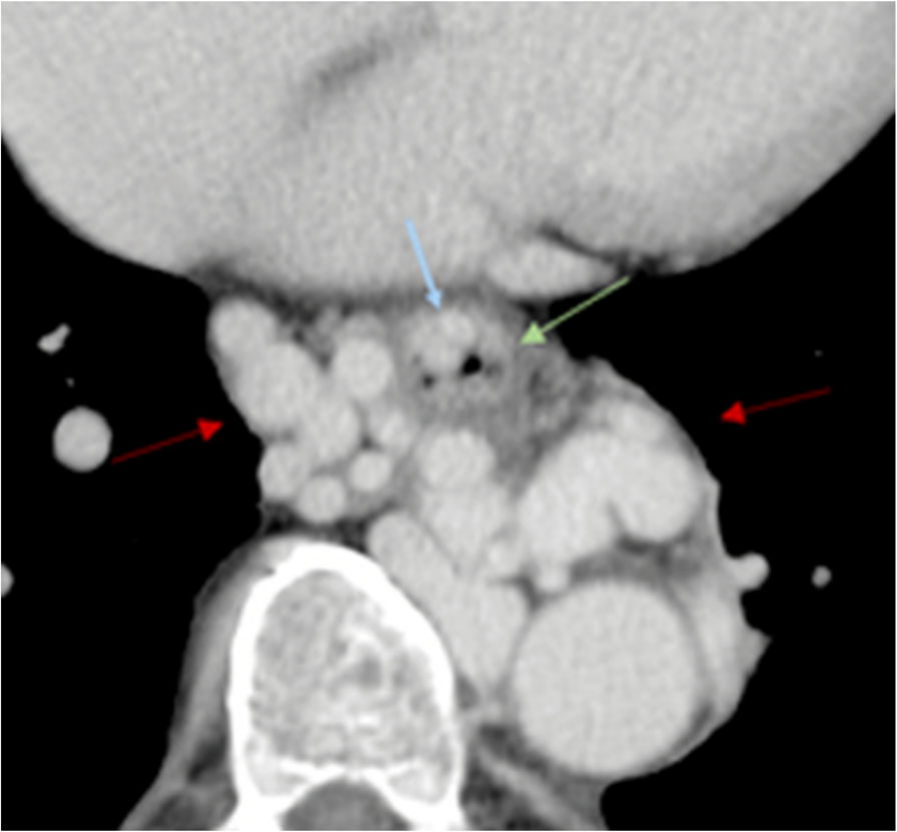
Esophageal and paraesophageal varices. Portal venous phase CT axial images show paraesophageal varices (red arrow) in the mediastinum, appearing as dilated tortuous veins surrounding the outer surface of the esophageal inferior third (green arrow), and esophageal varices (blue arrow), presenting as submucosal varicose veins within the esophagus wall

Esophageal varices are of major clinical importance because they are a frequent source of gastrointestinal bleeding (Fig. 24). Endoscopy is more sensitive in the diagnosis of esophageal mucosal varices but CT shows to better advantage the paraesophageal varices. Both venous plexuses communicate via perforating veins, crossing the muscular layer of the esophageal wall.

Paraumbilical vessels are an accepted pathway to decompress the PVS because they are not associated with gastrointestinal bleeding. The most common drainage pattern of paraumbilical veins is through the epigastric veins into the external iliac veins (Fig. 19).

**Fig. 25:**
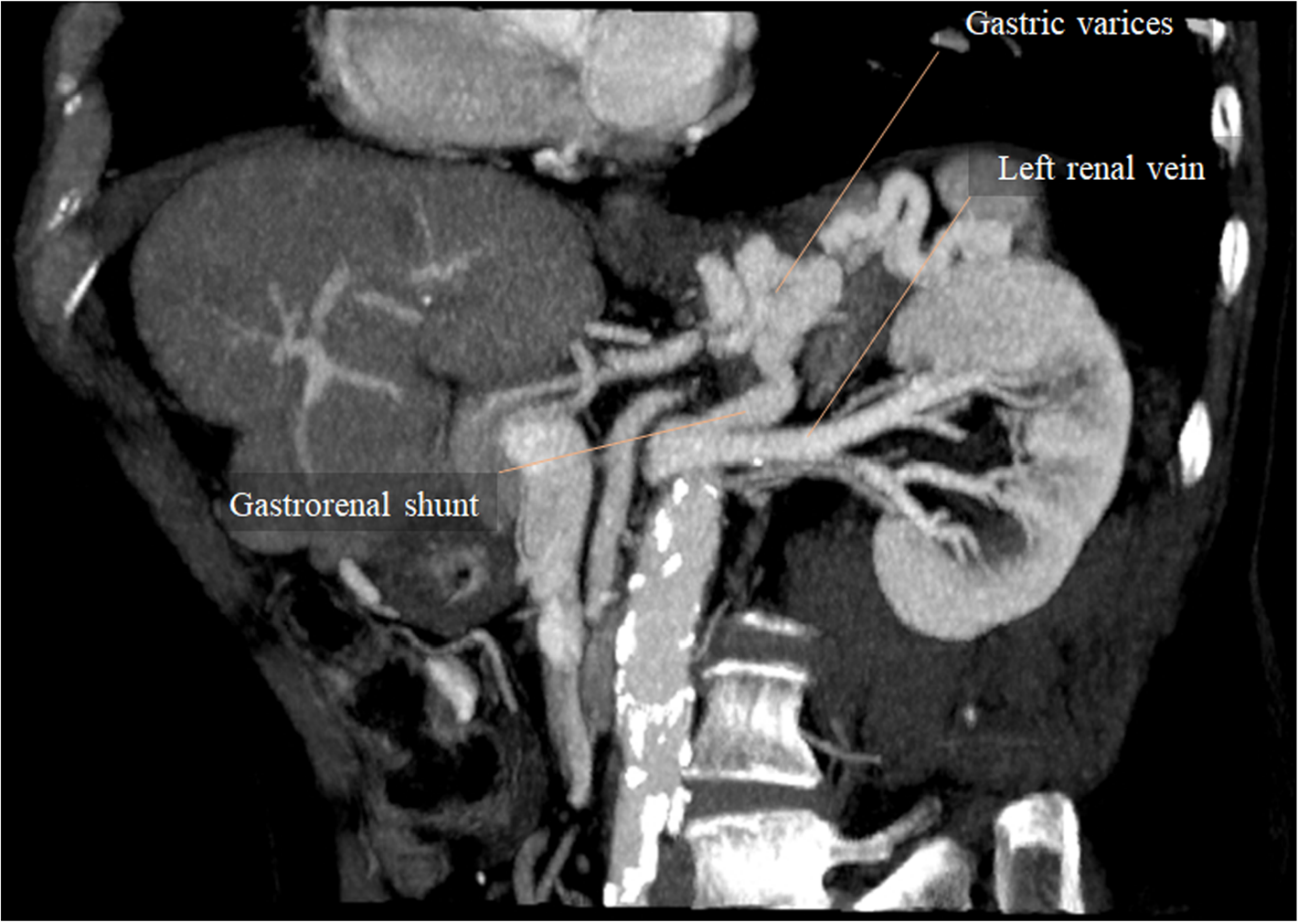
Gastrorenal shunt. MPR image from portal venous phase CT at the level of the left kidney shows a tortuous and high-caliber gastrorenal shunt, running from gastric varices to the left renal vein. Note the increased diameter of the left renal vein downstream shunt confluence, compared to the normal sized left renal vein segment upstream

Collateral vessels draining into the left renal vein are fairly common, and they are not associated with gastrointestinal bleeding either. However, enlarged shunts are associated with hepatic encephalopathy. A common feature depicted at cross-sectional imaging is an enlarged left renal vein and IVC dilatation at the level of the left renal vein when in the presence of a splenorenal/gastrorenal shunt (Fig. 25).

**Fig. 26:**
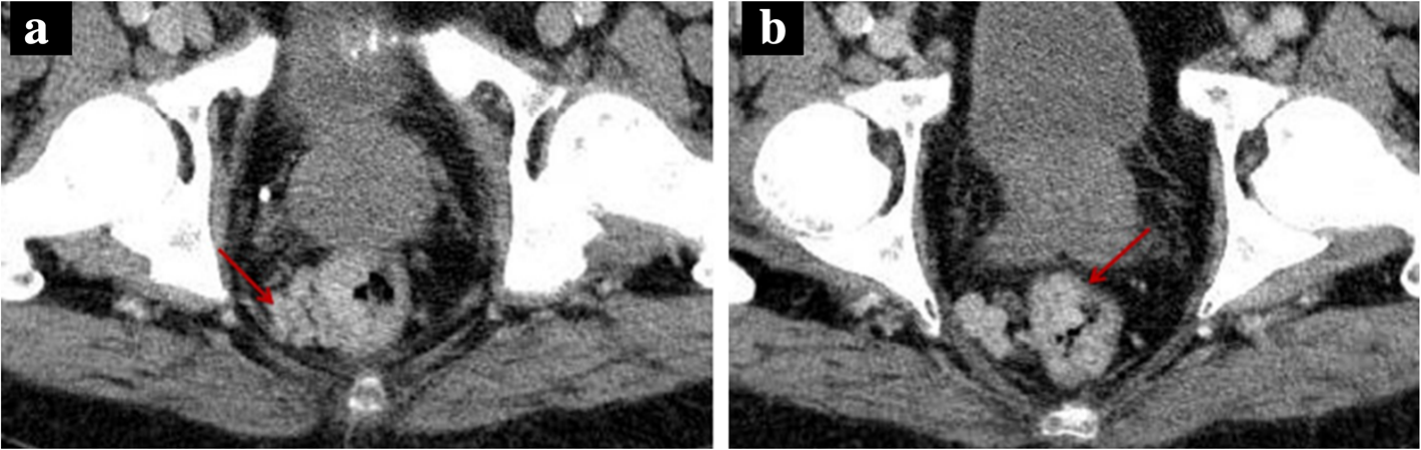
Anorectal/hemorrhoidal varices. Portal venous phase CT images showing a variceal network along, around and within the anorectal wall. In this case, we can individualize ingurgitated tortuous vessels (red arrows), but sometimes thickening and hyperenhancement are all we can see

Inferior mesenteric collateral vessels are less frequent but of great importance because of their association with rectal bleeding, since the PVS (superior hemorrhoidal vein) and the systemic venous circulation (middle and inferior hemorrhoidal veins) connect via the hemorrhoidal plexus (Fig. 26).

**Fig. 27:**
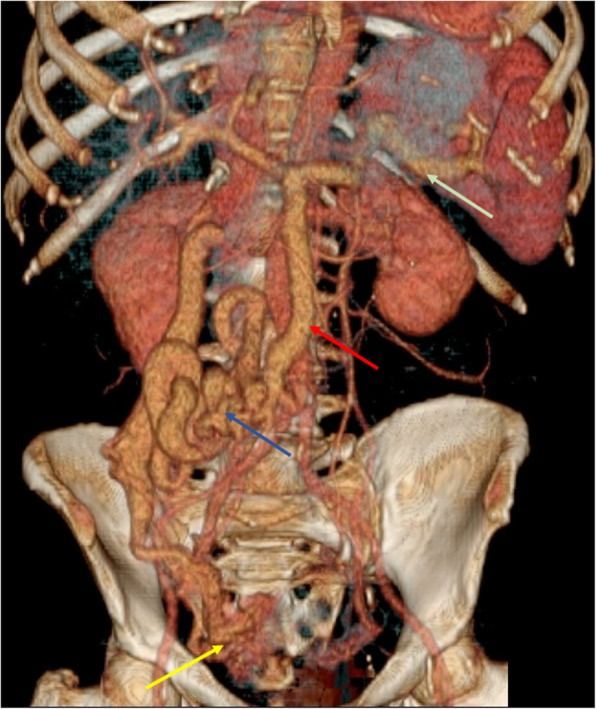
Retroperitoneal varices. VRT image from portal venous phase CT shows an increase in size of SV (green arrow) and SMV (red arrow), an extensive collateral network of varices fed by SMV (blue arrows), communicating with a huge-caliber right ovarian vein (yellow arrow), and giving rise to varices along the ovarian venous plexus

Mesocaval shunts are portosystemic collateral vessels between the SMV and IVC that are established through retroperitoneal veins, and are not associated with an increased risk of rectal bleeding (Fig. 27).

#### Iatrogenic abnormalities

Acquired intrahepatic portosystemic shunt can result from trauma, PH, and in the setting of transjugular intrahepatic portosystemic shunt (TIPS).

**Fig. 28:**
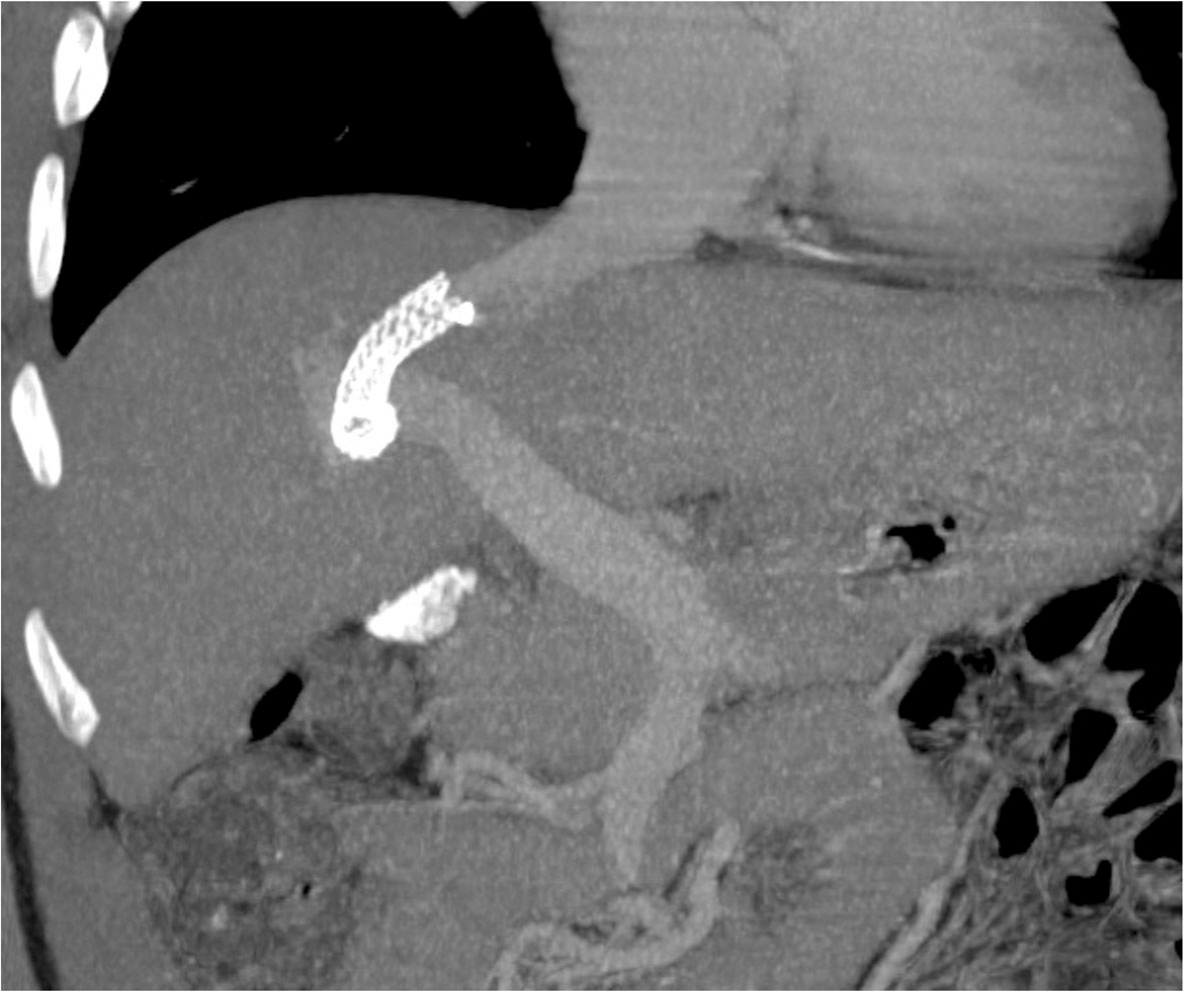
Transjugular intrahepatic portosystemic shunt. MIP image from portal venous phase CT shows a metallic stent connecting the PV with the hepatic vein. Note enlargement of the hepatic vein due to increased flow directly drained from the PVS to its lumen, skipping the hepatic parenchyma

The creation of a TIPS in the context of PH is one of the most common percutaneous interventional procedures involving PVS, where a parenchymal channel between a large hepatic vein and a major PV branch is created by inserting an expandable stent, in most cases connecting the right PV with the right or middle hepatic vein (Fig. 28).

**Fig. 29:**
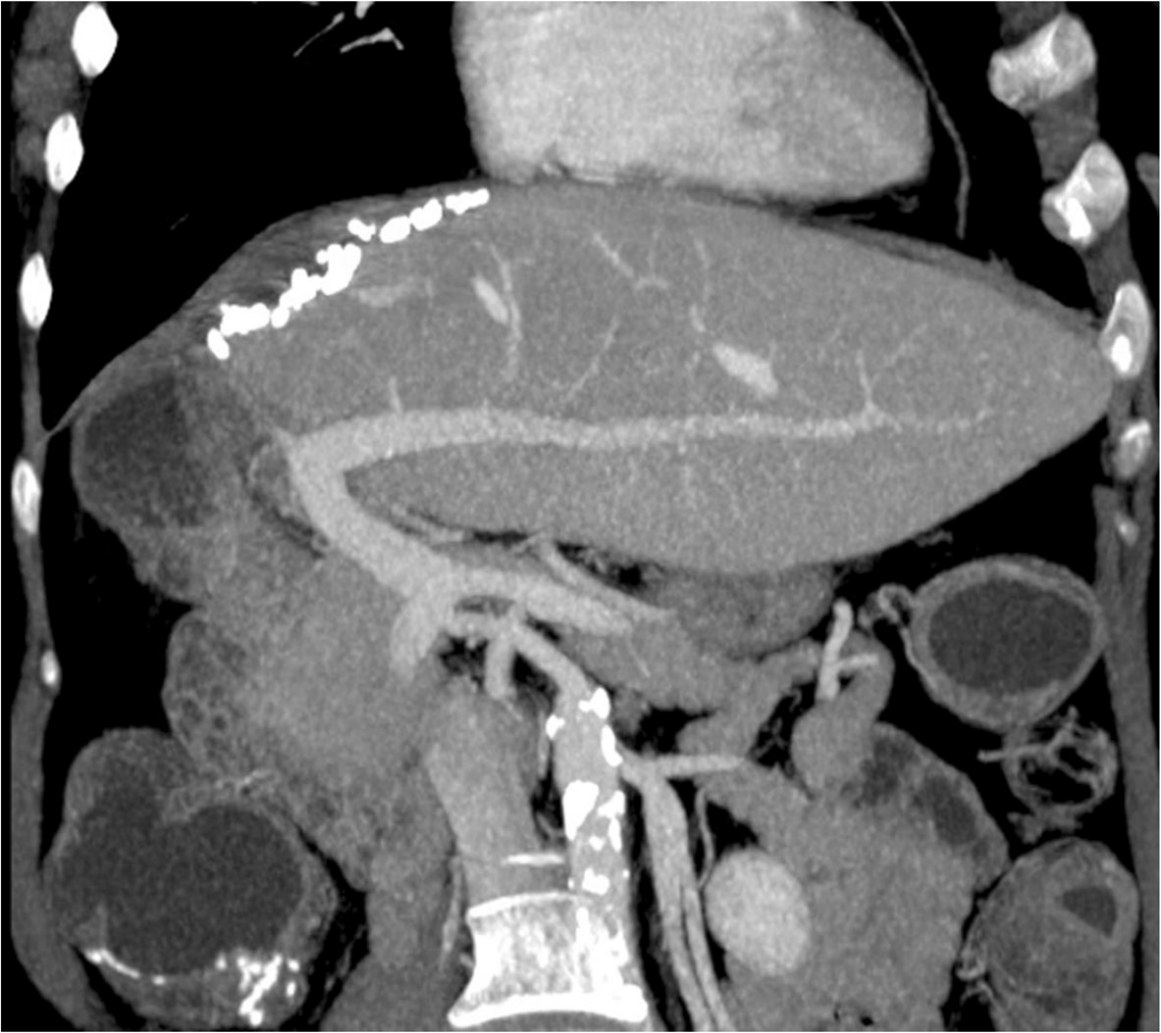
Liver surgery. MIP image from portal venous phase CT shows right hepatectomy findings: section and ligation of the right PV at its origin; shift of left liver toward the right with horizontalization of PV; prominent caliber of left PV and hypertrophy and rounded contours of the remaining liver

Other iatrogenic findings may result from post-surgical changes. Liver surgery mainly consists in anatomical-oriented resections, based on the intrahepatic distribution of portal branches and hepatic veins [19]. Segmentectomy always involves portal venous branches ligation and rearrange of the remaining system, conducing to an increased vessel caliber and/or adaptive shifting (Fig. 29).

## Conclusion

Understanding the embryology, normal anatomy, and anatomical variants of the PVS is important to accurately interpret abdominal findings. Knowledge of typical features of congenital and acquired PV pathologies allows the radiologist to make a confident diagnosis potentially impacting patient management.

## Abbreviations

IMV: Inferior mesenteric vein
MIP: Maximum intensity projection
MPR: Multiplanar reformation
OPV: Obliterative portal venopathy
PH: Portal hypertension
PV: Portal vein
IVC: Inferior vena cava
PVS: Portal venous system
SMV: Superior mesenteric vein
THED: Transient hepatic enhancement difference
TIPS: Transjugular intrahepatic portosystemic shunt
VRT: Volume rendering technique
